# Neutrophil S100A9 supports M2 macrophage niche formation in granulomas

**DOI:** 10.1016/j.isci.2023.106081

**Published:** 2023-01-31

**Authors:** Tatsuaki Mizutani, Toshiaki Ano, Yuya Yoshioka, Satoshi Mizuta, Keiko Takemoto, Yuki Ouchi, Daisuke Morita, Satsuki Kitano, Hitoshi Miyachi, Tatsuaki Tsuruyama, Nagatoshi Fujiwara, Masahiko Sugita

**Affiliations:** 1 Laboratory of Cell Regulation, Institute for Life and Medical Sciences, Kyoto University, Kyoto, Japan; 2 Laboratory of Cell Regulation and Molecular Network, Graduate School of Biostudies, Kyoto University, Kyoto, Japan; 3 Center for Bioinformatics and Molecular Medicine, Graduate School of Biomedical Sciences, Nagasaki University, Nagasaki, Japan; 4 Laboratory of Immune Regulation, Institute for Life and Medical Sciences, Kyoto University, Kyoto, Japan; 5 Reproductive Engineering Team, Institute for Life and Medical Sciences, Kyoto University, Kyoto, Japan; 6 Department of Drug Discovery Medicine, Graduate School of Medicine, Kyoto University, Kyoto, Japan; 7 Department of Food and Nutrition, Tezukayama University, Nara, Japan

**Keywords:** Cancer, Components of the immune system, Immunology, Molecular biology

## Abstract

Mycobacterium infection gives rise to granulomas predominantly composed of inflammatory M1-like macrophages, with bacteria-permissive M2 macrophages also detected in deep granulomas. Our histological analysis of *Mycobacterium bovis* bacillus Calmette-Guerin-elicited granulomas in guinea pigs revealed that S100A9-expressing neutrophils bordered a unique M2 niche within the inner circle of concentrically multilayered granulomas. We evaluated the effect of S100A9 on macrophage M2 polarization based on guinea pig studies. S100A9-deficient mouse neutrophils abrogated M2 polarization, which was critically dependent on COX-2 signaling in neutrophils. Mechanistic evidence suggested that nuclear S100A9 interacts with C/EBPβ, which cooperatively activates the *Cox-2* promoter and amplifies prostaglandin E2 production, followed by M2 polarization in proximal macrophages. Because the M2 populations in guinea pig granulomas were abolished via treatment with celecoxib, a selective COX-2 inhibitor, we propose the S100A9/Cox-2 axis as a major pathway driving M2 niche formation in granulomas.

## Highlights

• Nuclear S100A9 interacts with C/EBPβ, which activates the Cox-2 gene promoter
• S100A9 upregulates prostaglandin E2 production, driving M2 polarization of macrophages
• The S100A9-Cox-2 axis is critical for M2 niche formation in granulomas

## Graphical abstract

**Figure:**
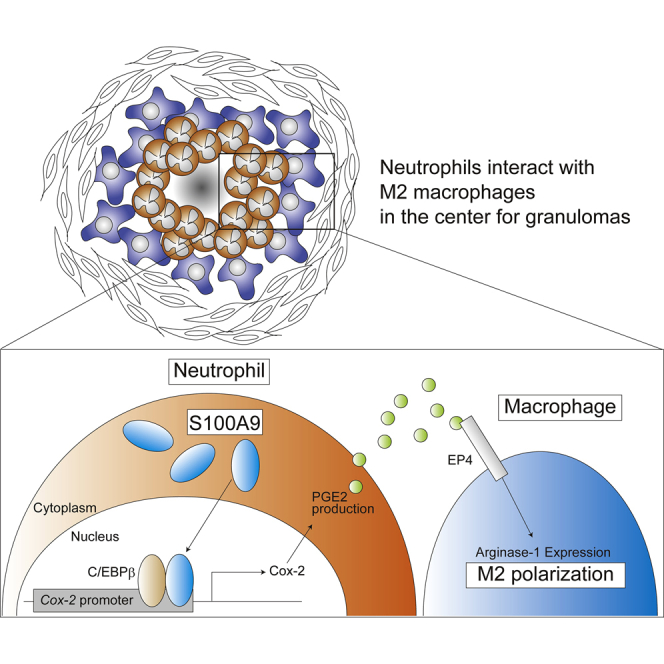

## Introduction

Immune cell interplay determines the extent of inflammation including its timing, intensity, and duration.^1^ Disruption of intercellular communication meant to attenuate inflammation causes various chronic diseases. Therefore, the identification of cellular and molecular inflammatory factors subject to tight regulation is biologically and clinically relevant.

Granulomas are characteristic globular structures comprising densely packed macrophages and represent a hallmark of several chronic inflammatory diseases, including tuberculosis, sarcoidosis, and Crohn disease, among other human granulomatous disorders.^2^^,^^3^^,^^4^^,^^5^^,^^6^ Histological analyses have revealed that in addition to other immune cell types, including T- and B-lymphocytes and neutrophils, a prominent cell fraction of persistently activated macrophages, called epithelioid cells, accumulated within granulomas.^7^ Intercellular interactions within granulomas drive effective inflammatory responses against pathogens or contaminants; however, such inflammation persists over prolonged periods of time.^7^^,^^8^ Therefore, elucidating granulomatous cell interactions that influence the magnitude of inflammation is critical for understanding granuloma development.

Macrophages within a granuloma are rather heterogeneous.^9^^,^^10^ In addition to the central population of inflammatory M1-like epithelioid cells, granulomas comprise anti-inflammatory macrophages of the M2 phenotype.^11^ Owing to their phenotypic plasticity, macrophages are subdivided into M1 and M2 according to their ability to differentiate following stimulation with cytokines and microbial molecules, with L-arginine-hydrolyzing enzyme arginase-1 (Arg1) being specifically expressed in M2 macrophages.^12^^,^^13^ In the case of *Mycobacterium tuberculosis* infection, anti-inflammatory M2 macrophages promoted bacterial survival by suppressing NO production through Arg1-dependent competitive substrate depletion.^14^^,^^15^ Thus, granulomatous M2 macrophages form a conducive environment for bacteria that cause chronic inflammation; however, the molecular pathway involved in M2 induction is not fully understood. In particular, functional cell-cell communication and associated molecules regulating macrophage M2 polarization in granulomas remain elusive.

We have previously established a *Mycobacterium bovis* bacillus Calmette-Guerin (BCG)-induced lung granuloma model in guinea pigs, which closely resembled human tuberculosis granulomas.^16^ We demonstrated the specific accumulation of S100A9 (A9) at the necrotic core of granulomas, controlling initial granuloma formation. A9 is expressed at low levels in monocytes and macrophages, whereas it is present at high levels within neutrophils, where it is found only as a heterodimer complexed with S100A8 (A8).^17^^,^^18^^,^^19^ Owing to the various functions of A9 in inflammation mediated through autocrine and paracrine interactions with other cells, including neutrophils, macrophages, and keratinocytes, both the inflammatory and anti-inflammatory effects of A9 have been reported in A9-deficient mice.^17^^,^^18^^,^^20^^,^^21^^,^^22^^,^^23^ Considering this multifunctional nature of A9 in neutrophils, this study aimed to investigate its effect on granuloma macrophage polarization.

## Results

### Association of S100A9-expressing neutrophils with M2 macrophages in granulomas

**Figure 1:**
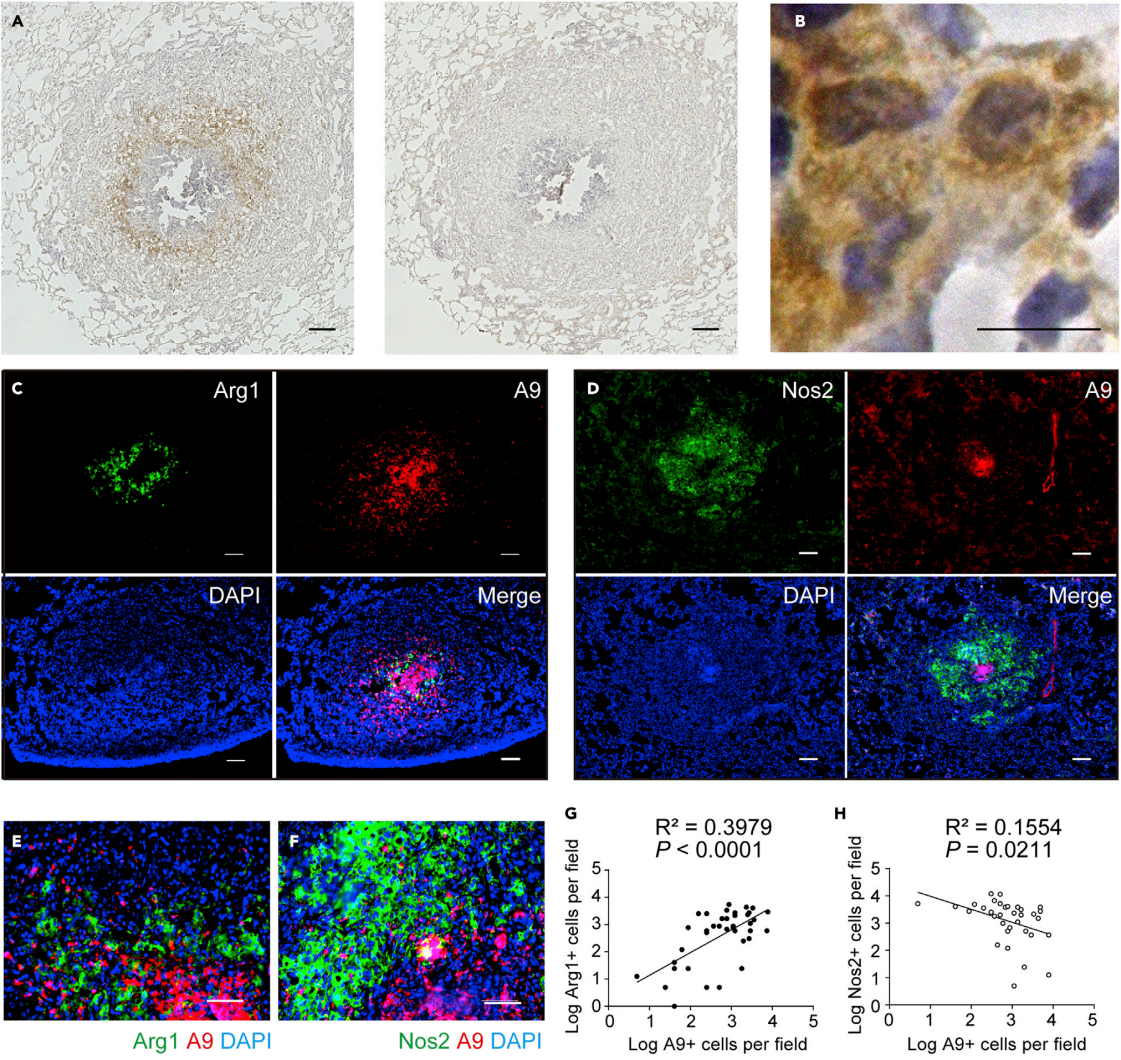
Arg1^+^ macrophages accumulated in the central area of granulomas (A) Granulomas from BCG-challenged guinea pig lungs were labeled with anti-Arg1 antibody (Ab) (left panel) or isotype control Ab (right panel), and horseradish peroxidase-3,3′-diaminobenzidine–based detection was performed. Granulomas were counterstained with hematoxylin. Scale bars, 100 μm. (B) A magnified view of Arg1^+^ cells in (A) shows typical macrophage morphology. Scale bars, 10 μm. (C and D) Representative fluorescence microscopy images of the granulomas labeled with anti-Arg1 (green) and anti-A9 Abs (red) (C) or with anti-Nos2 (green) and anti-A9 Abs (red) (D). Scale bars, 100 μm. (E and F) Magnified (40×) images of granuloma sections co-stained for Arg1^+^ M2 macrophages (green) and A9^+^ neutrophils (red) (E) or for Nos2^+^ cells (green) and A9^+^ neutrophils (red) (F). Nuclei were counterstained with DAPI (blue). Scale bars, 100 μm. (G and H) Scatterplots for the number of Arg1^+^ and A9^+^ cells (•, G) or Nos2^+^ and A9^+^ cells (○, H) in the same microscopic field (100×). Data are pooled from three independent experiments (n = 6 guinea pigs per group). Statistical analysis included simple linear regression. Significant p values in (G) and (H) reflect significantly non-zero slopes determined via linear regression.

Granulomas contain multilayered epithelioid M1-like cells, which serve as cellular and immunological barriers for the internal confinement of mycobacteria.^6^ However, pathogenic mycobacteria often persist in the central part of granulomas, suggesting that unique microenvironments are formed, favoring bacterial survival. We previously showed that A9-expressing neutrophils accumulate at the core of granulomas and predicted that macrophages localized at the M1-neutrophil junction may undergo functional transformation. Indeed, we found that mononuclear cells surrounding the neutrophil core prominently expressed Arg1, an enzyme that counteracts Nos2 expression in M1 macrophages (Figure 1A). Furthermore, Arg1^+^ cells were large in size, with a long diameter >20 μm, and showed typical horseshoe-shaped nuclei with pale hematoxylin staining (Figure 1B). These histological observations suggest that Arg1^+^ cells are M2-polarized macrophages. As shown in Figure 1C, Arg1^+^ macrophages were prominently confined to the center of granulomas (upper left), and A9^+^ neutrophils were specifically concentrated at the granuloma core (upper right). In contrast, Nos2^+^ macrophages were present throughout the granulomas (Figure 1D). These observations suggest a specific proximal location for Arg1^+^ and A9^+^ neutrophils and the inner A9^+^ cluster spatially discriminating Nos2^+^ M1 (Figures 1E and 1F). The number of A9^+^ neutrophils positively correlated with that of Arg1^+^ macrophages (*R*^2^ = 0.3979, p < 0.0001; Figures 1E and 1G) but negatively correlated with that of Nos2^+^ macrophages (R^2^ = 0.1554, p = 0.0211; Figures 1F and 1H). These correlations indicate that granulomatous macrophages are favorably polarized into M2 macrophages within A9^+^ neutrophil-rich areas.

### Neutrophil A9 facilitates M2 macrophage polarization

**Figure 2:**
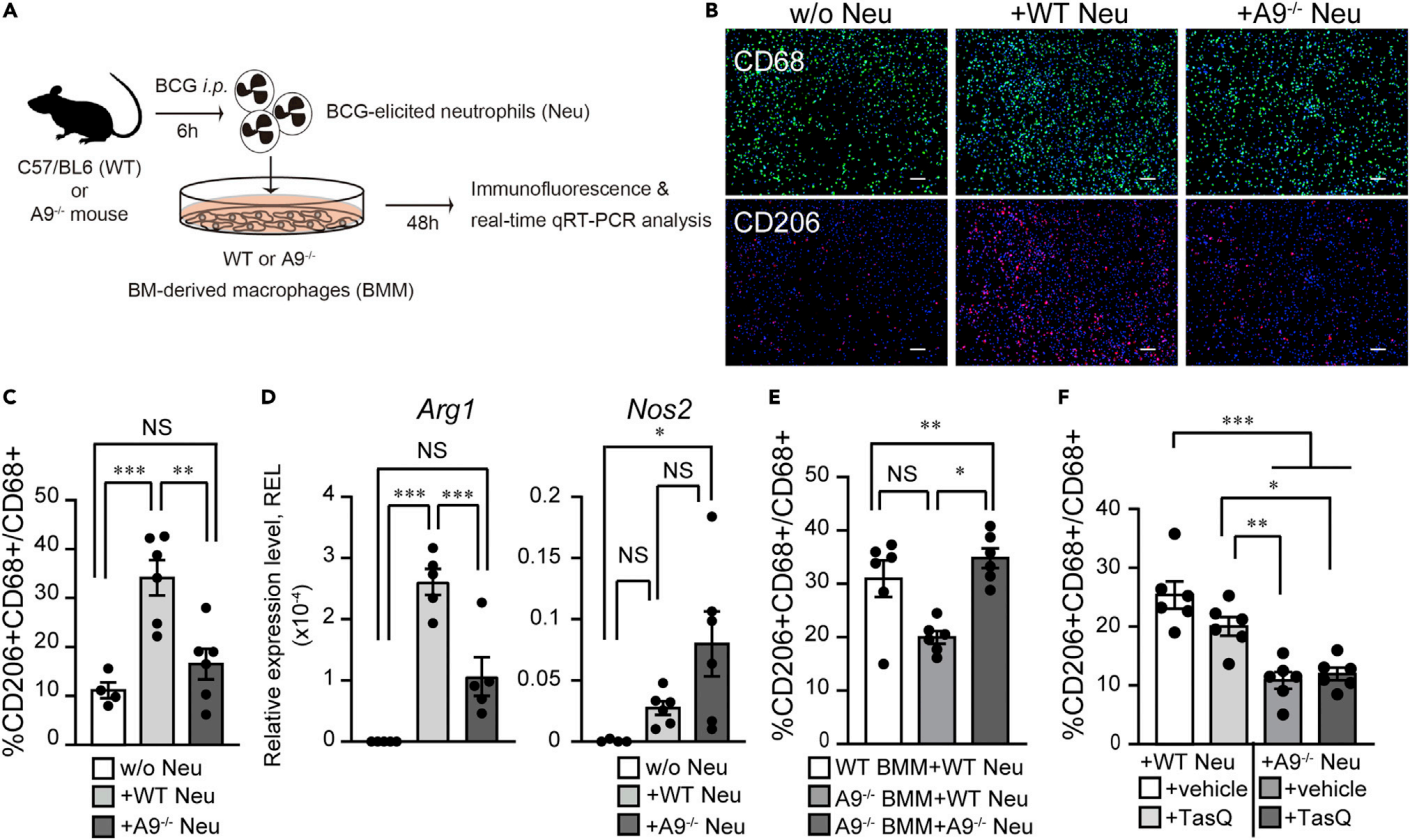
The BCG-elicited A9^−/−^ neutrophils failed to polarize M0-BMMs into M2 state (A) A schematic diagram of co-culture experiments with WT or A9^−/−^ bone marrow macrophages (BMMs) and WT or A9^−/−^ BCG-elicited neutrophils (Neu) obtained following BCG inoculation. The BCG-elicited, highly purified neutrophils were added to BMMs. At 48 h after co-culture, immunostaining and real-time qRT-PCR were performed. (B) Representative immunofluorescence images of BMMs co-cultured with BCG-elicited WT, A9^−/−^ Neu, or without Neu (w/o Neu) for 48 h; anti-CD68 (green) and anti-CD206 (red) Abs are shown. Scale bars, 10 μm. (C) Percentage of CD206^+^ cells among CD68^+^ total macrophages from WT-BMMs co-cultured with WT, A9^−/−^ Neu, or without Neu. (D) Expression of *Arg1* and *Nos2* mRNAs in BMMs co-cultured with or without Neu for 48 h. Quantitative RT-PCR values were normalized to the expression of the *β2-microglobulin* (*β2m*) gene. (E) Percentage of CD206^+^ cells among CD68^+^ total macrophages from A9^−/−^ or WT BMMs co-cultured with or without Neu. (F) Percentage of CD206^+^ cells among CD68^+^ total macrophages from BMMs co-cultured with Neu treated with or without tasquinimod (TasQ). Data are pooled from two independent experiments (n = 4–6 mice per group). Data are expressed as the mean ± SEM in (C–F). ∗∗∗p < 0.0005, ∗∗p < 0.005, ∗p < 0.05; ANOVA and post hoc Tukey-Kramer tests.

Based on the results of guinea pig histology, we speculated that BCG-elicited neutrophils are involved in M2 induction. To test this hypothesis, we generated A9-deficient mice (hereafter A9^−/−^) using the CRISPR/Cas9 system and obtained A9^−/−^ BCG-elicited neutrophils. Neutrophils were enriched via magnetic sorting from the BCG-injected peritoneal cavity (Figure S1) and added above the monolayer culture of naive bone marrow–derived macrophages (BMMs) (Figure 2A). Neutrophils were directly associated with macrophages for the initial 24 h, whereafter macrophages were the dominant population in the cell culture. To evaluate the impact of neutrophil-induced M2 polarization, we performed an immunofluorescence analysis of the mannose receptor CD206, a practical murine M2 marker (Figures 2B and S2).^24^^,^^25^ As BMMs were independently cultured for 2 days in the absence of neutrophils, CD206^+^ M2 was detected in approximately 10% of CD68^+^ pan-macrophages. In the presence of BCG-elicited wild-type (WT) neutrophils, the number of CD206^+^ M2 macrophages increased 3-fold compared with the control (Figures 2B and 2C). Strikingly, BMMs co-cultured with A9^−/−^ neutrophils exhibited a lack of CD206^+^ macrophage induction, with no significant difference in number compared to BMMs without neutrophil addition (Figure 2C). We simultaneously monitored *Arg1* and *Nos2* expression in the co-cultured samples using qRT-PCR analysis. As shown in Figure 2D, BMMs co-cultured with WT or A9^−/−^ neutrophils had more than 10-fold increase in *Arg1* and *Nos2* expression than the BMMs control. BMMs co-cultured with A9^−/−^ neutrophils exhibited significantly lower Arg1 expression than BMMs co-cultured with WT neutrophils. *Nos2* was upregulated in both BMMs in the presence of A9^−/−^ and WT neutrophils. In addition, the M2-related genes such as *Fizz1* and *IL-10* were downregulated in BMMs co-cultured with A9^−/−^ neutrophils compared with those in BMMs co-cultured with WT neutrophils (Figure S3). These results indicate that BCG-elicited neutrophils potentiated the polarization of BMMs into M2 state.

Because A9 is expressed in macrophages, albeit at low levels, A9^−/−^ macrophages were employed in the co-culture system to test whether A9 expressed on macrophages regulates M2 polarization in an autocrine manner. A significant increase in the number of CD206^+^ M2 macrophages was detected in A9^−/−^ BMMs co-cultured with WT compared to those with A9^−/−^ neutrophils. Importantly, the CD206^+^ M2 cell numbers in A9^−/−^ BMMs co-cultured with WT neutrophils were comparable to those in WT BMMs with WT neutrophils (Figure 2E). These results suggest that A9 expressed in neutrophils, rather than macrophage-expressing A9, is critical for the M2 polarization of neutrophil-associated macrophages.

A9 forms an active biological heterodimer with A8 (calprotectin) and is released from inflamed and damaged cells.^26^ Extracellular calprotectin functions as an alarmin that activates the innate immune system. Some alarmin receptor signaling pathways are involved in the regulation of M1-M2 polarization.^14^^,^^27^ Tasquinimod (TasQ) is a small-molecule inhibitor of the heterodimerization between A9 and A8, thus shutting down alarmin signaling.^28^ Next, we tested whether the alarmin function of A9 affects M2 polarization in a TasQ-treated BMMs-neutrophil culture experiment. Immunofluorescence results revealed that TasQ treatment led to a slight decrease in the number of CD206^+^ cells compared to DMSO treatment (+vehicle) (Figure 2F). Neither A9^−/−^ neutrophils treated with TasQ nor vehicle-treated controls induced CD206^+^ macrophages in the co-cultured BMMs. Similarly, qRT-PCR analysis showed a significant upregulation of *Arg1* in BMMs co-cultured with both vehicle- and TasQ-treated neutrophils (Figure S4). *Nos2* expression was inhibited by TasQ under both WT and A9^−/−^ neutrophil co-culture with BMMs. This inhibitory effect of TasQ may be associated with the TNFα induced by macrophage-derived A9.^29^ TasQ had a minimal inhibitory effect on *in vitro* M2 polarization, suggesting that the extracellular alarmin function of A9 is unlikely to play a critical role in neutrophil-dependent M2 polarization. Furthermore, casein-induced neutrophils, categorized as non-stimulated neutrophils, exhibited a lower capacity for inducing M2 macrophages (Figure S5). Taken together, co-culture experiments showed that neutrophils activated by BCG have the critical ability to polarize macrophages into M2 in an A9-dependent manner.

### Effect of A9 on *in vivo* M1/M2 polarization influences bacterial control

Our observations suggested that neutrophils activated during BCG infection may continuously induce M2-polarized macrophages. We observed rapid accumulation of neutrophils in the peritoneal cavity of mice at 6 h post-BCG-injection (pBi), prior to macrophage dominance; at 7 days pBi, neutrophils (CD11b^+^CD11c^−^F4/80^-^Ly6G^+^) were still accumulating (Figure S6). Continuous cellular interplay between neutrophils and macrophages in the abdominal environment for 1 week can be assumed as similar to the process of granuloma formation. BCG-injected A9^−/−^ and WT mice showed no significant difference in the frequencies or absolute number of macrophages and neutrophils accumulated within their peritoneal cavity throughout the indicated time course (Figure S6), suggesting that A9 deficiency did not affect cell recruitment to the lesion.

**Figure 3:**
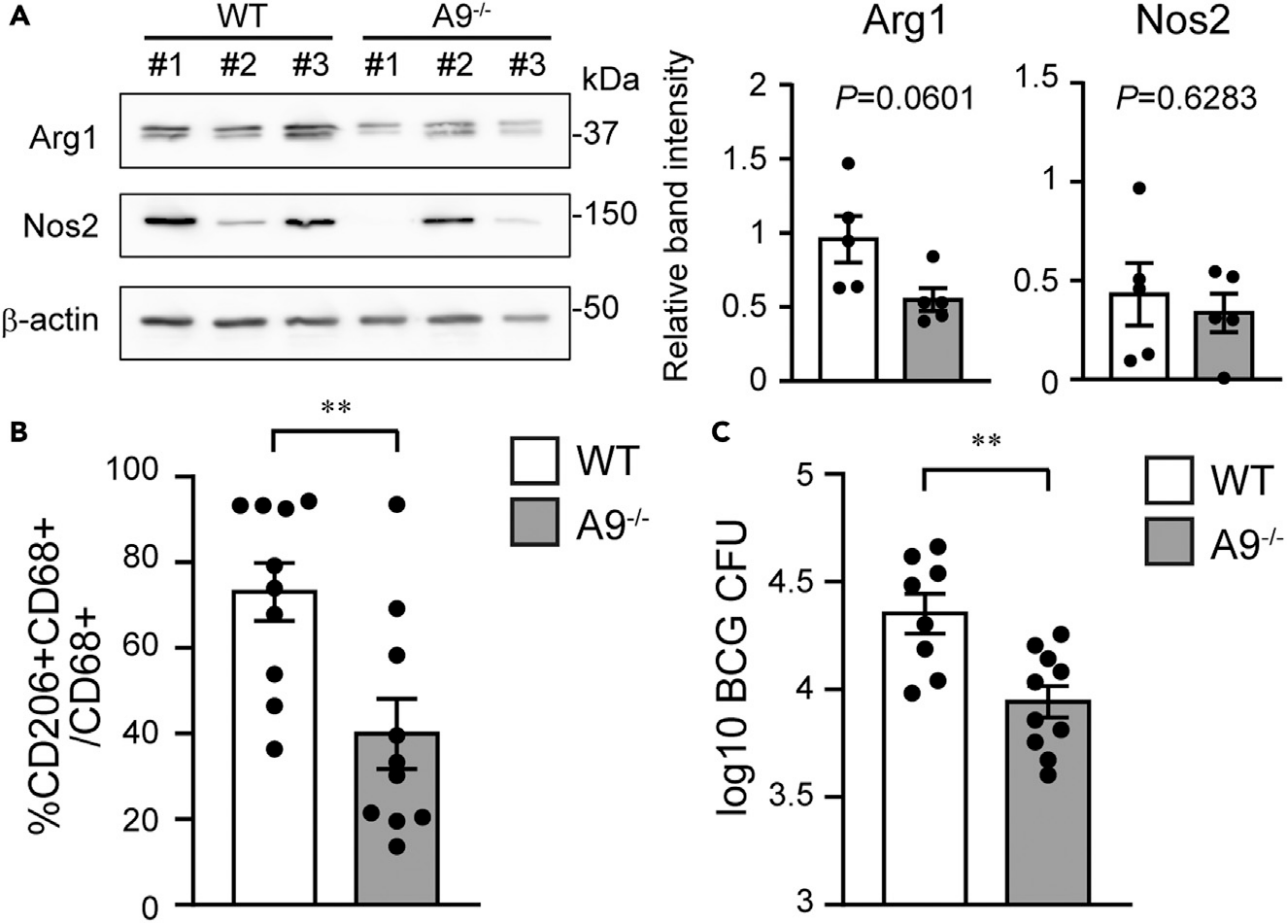
Imbalance of M1/M2 polarization and enhanced bacterial killing in A9^−/−^ mice (A) Total cell lysates from BCG-elicited peritoneal macrophages were resolved on SDS-PAGE, and western blotting was conducted with Abs against Arg1 and β-actin. Quantitative data on band intensities pooled from two independent experiments (right). (B) Five days after BCG inoculation, the peritoneal macrophages from WT and A9^−/−^ mice were stained with anti-CD68 (green) and anti-CD206 (red) Abs. Percentage of CD206^+^ cells among CD68^+^ total macrophages from WT (open) and A9^−/−^ (gray) mice. (C) The CFU of BCG in the peritoneal cavity of WT and A9^−/−^ mice at 1 week after BCG infection is shown. Data were from 1 representative experiment of two independent experiments (A, left panel) or n = 3–5 mice per group (A, right panel; n = 6, B; and C, n = 10). Data are shown as mean ± SEM. ∗∗p < 0.005, Welch’s *t*-test.

To investigate A9-dependent M2 regulation, we first measured *Arg1* expression in BCG-elicited WT or A9^−/−^ peritoneal macrophages at 5 days pBi. The level of Arg1, but not of Nos2, in macrophages decreased in A9^−/−^ mice compared with that in the control, suggesting that A9^−/−^ macrophages have a similar *Nos2* expression but reduced *Arg1* expression when compared to WT macrophages (Figure 3A). A total of 66.0 ± 5.65% of WT CD68^+^ macrophages expressed CD206 (Figure 3B). Consistent with insufficient *Arg1* expression in A9^−/−^ mice, the number of CD206-expressing macrophages was strongly reduced in A9^−/−^ mice (41.6 ± 8.89%, Figure 3B). Furthermore, we detected decreased levels of Fizz1, Ym1, and IL-10 in A9^−/−^ macrophages compared with those in WT macrophages (Figure S7). These results support the requirement of A9 for M2 induction during *in vivo* BCG infection.

Immunofluorescence analysis detected BCG-infected macrophages at the indicated times (Figure S8). The number of viable bacteria markedly decreased in A9^−/−^ mice compared with that in WT mice, reflecting heightened mycobacterial suppression under lower M2 polarization in A9^−/−^ mice (Figure 3C).

### COX-2 induction by neutrophils is critical for M2 polarization

As A9 regulates the transcription of numerous genes,^20^^,^^30^ we hypothesized that A9 influences neutrophilic gene expression associated with M2 induction. To explore the molecular basis of A9-dependent neutrophil-driven M2 polarization during BCG infection, we performed an RNA-seq analysis of peritoneum-recruited mature neutrophils from WT and A9^−/−^ mice after BCG infection. In addition to gene sets from the BCG-infected sample, we prepared casein-induced neutrophils from WT and A9^−/−^ mice as basal control groups. RNA-seq identified 813 differentially expressed genes (DEGs) following BCG infection and 786 DEGs under casein-elicited conditions. Of these, 340 were downregulated and 473 were upregulated in A9^−/−^ neutrophils compared to those in WT neutrophils following BCG injection. Based on DAVID^31^ GO and pathway enrichment analyses, lipid-metabolism-related genes were among BCG-elicited DEGs (Tables S1 andS2). In cellular lipid metabolism, arachidonic acid (AA) metabolism is essential for macrophage polarization.^32^ Therefore, we focused on the factors controlling AA metabolism and prostaglandin endoperoxide synthetase 2 (Ptgs2; also called Cox-2), which is required for prostanoid synthesis.

**Figure 4:**
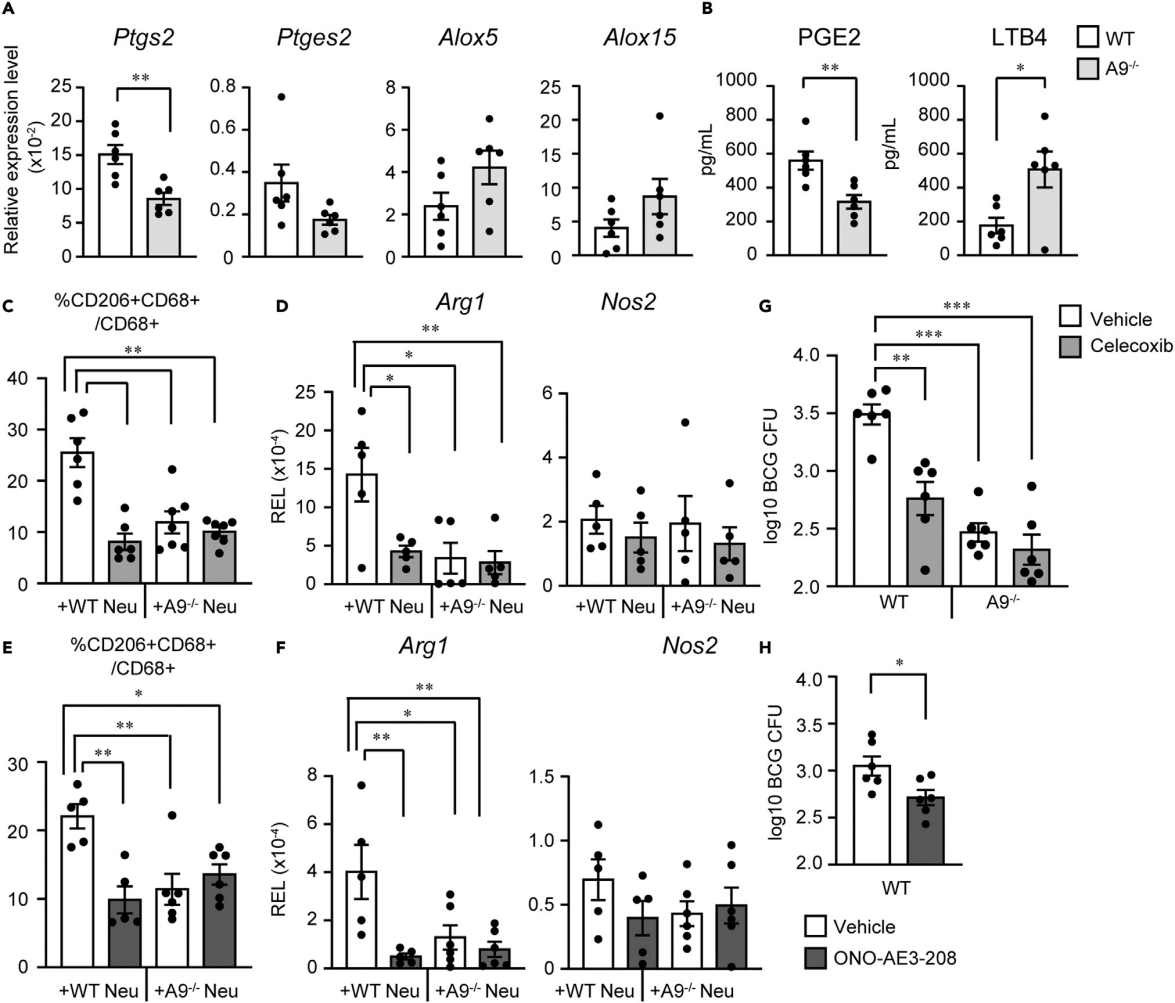
Neutrophil A9-dependent COX-2 induction for macrophage M2 polarization (A) Relative expression levels of *Ptgs2*, *Ptges2*, *Alox5*, and *Alox15* mRNA from BCG-elicited WT or A9^−/−^ neutrophils were measured via the delta CT method and normalized to *β2m* expression levels. (B) Amount of PGE2 or LTB4 in the 18-h culture supernatant of BCG-elicited neutrophils from WT and A9^−/−^ mice. (C) Percentage of CD206^+^ cells among CD68^+^ total macrophages from BMMs co-cultured with Neu pre-treated with the COX-2-specific inhibitor celecoxib. (D) mRNA Expression of *Arg1* and *Nos2* in BMMs co-cultured with celecoxib-treated Neu was analyzed via quantitative real-time PCR. (E) Percentage of CD206^+^ cells among CD68^+^ total macrophages from WT BMMs treated with or without EP antagonist ONO-AE3-208 before the addition of Neu. (F) *Arg1* and *Nos2* mRNA induction in BMMs, as indicated in (E), was analyzed via quantitative real-time PCR. (G) CFU of BCG in the peritoneal macrophages of WT or A9^−/−^ mice treated with celecoxib (light gray) or vehicle (open) at 1 week after BCG infection is shown. (H) CFU of BCG in the peritoneal macrophages of ONO-AE3-208-treated (dark gray) and vehicle-treated (open) WT mice is shown. Data were pooled from two independent experiments with three mice per experimental group (A, B, G, and H). Data are shown as mean ± SEM. ∗∗∗p < 0.0005, ∗∗p < 0.005, ∗p < 0.05, Welch’s *t*-test (A, B, G, and H) or ANOVA and post hoc Tukey-Kramer test (C–F).

Subsequently, we confirmed that the upregulation of Ptgs2 in BCG-elicited WT neutrophils was diminished in A9^−/−^ mice (Figure 4A). He et al.^20^ reported that A9 is involved in the upregulation of prostaglandin E synthetase (Ptges), which catalyzes the synthesis of the prostaglandin E2 (PGE2). We detected significantly lower *Ptges2* expression in A9^−/−^ neutrophils than in WT controls (Figure 4A). Conversely, the expression of leukotriene synthesis-related enzymes Alox5 and Alox15 was upregulated in A9^−/−^ neutrophils compared to that in WT neutrophils albeit without significant difference (Figure 4A). Consistent with this, BCG-elicited A9^−/−^ neutrophils had a significantly lower PGE2 production but a higher leukotriene B4 (LTB4) levels than the control (Figure 4B). These results suggest that A9 in neutrophils is essential for the upregulation of Ptgs2, followed by increased PGE2 production.

We investigated the necessity of the COX-2-PGE2 axis in M2 polarization of macrophages. The number of CD206^+^ M2 macrophages was reduced in the presence of celecoxib-treated neutrophils when compared to that in the presence of vehicle-treated controls (Figure 4C). Notably, the number of CD206^+^ cells among BMMs induced by either WT neutrophils (+celecoxib) or A9^−/−^ neutrophils (+vehicle) was comparable (Figure 4C). CD206^+^ populations were not further reduced in BMMs co-cultured with celecoxib-treated neutrophils when compared to those co-cultured with vehicle-treated A9^−/−^ neutrophils (Figure 4C). Consistently, celecoxib-treated neutrophils did not significantly upregulate the expression of Arg1 or affected Nos2 expression in macrophages (Figure 4D). These results suggest that neutrophil COX-2 is critical for the emergence of CD206^+^ M2, but not for M1 macrophages, which was almost entirely dependent on the A9 protein.

The A9/Cox-2 pathway promotes PGE2 production in neutrophils. PGE2 binds to cognate G-protein receptors EP1, EP2, EP3, and EP4 to exert its biological effects.^33^ BMMs were pre-treated with selective antagonists of the four known receptors for PGE2, followed by the addition of BCG-elicited WT neutrophils. Among these antagonists, the EP4 antagonist ONO-AE3-208^34^ specifically inhibited the induction of CD206^+^ M2 macrophages (Figure S9). We confirmed that compared to the vehicle treatment, ONO-AE3-208 treatment significantly reduced the number of CD206^+^ M2 co-cultured with WT neutrophils (Figure 4E). There was no significant difference in the number of CD206^+^ macrophages between A9^−/−^ neutrophils plus BMMs treated with ONO-AE3-208 and those without ONO-AE3-208. In addition, the number of CD206^+^ cells in these BMM groups was comparable with that of BMMs treated with ONO-AE3-208 and co-cultured with WT neutrophils. A decrease in CD206^+^ cells was supported by qRT-PCR results for *Arg1* expression (Figure 4F). In addition, the induction of Nos2^+^ M1 macrophages was not affected by ONO-AE3-208 treatment of WT BMMs co-cultured with WT or A9^−/−^ neutrophils (Figure 4F). These findings indicate that PGE2 production in BCG-elicited neutrophils is required for M2 polarization via EP4.

As the *in vitro* pharmacological blockade of neutrophilic COX-2 signaling inhibited M2 polarization, we investigated whether manipulating AA metabolism would influence host defense against BCG infection. The number of viable intracellular bacteria after BCG intraperitoneal infection decreased in the celecoxib-treated group compared to that in the control (Figure 4G). Of note, we found no significant difference in colony-forming unit (CFU) between celecoxib- and vehicle-treated A9^−/−^ mice (Figure 4G), suggesting that the bactericidal activity of A9 was almost entirely dependent on COX-2 signaling.

We also evaluated the effect of ONO-AE3-208 in BCG-injected mice. ONO-AE3-208 treatment decreased BCG CFUs (Figure 4H), suggesting that the PGE antagonist enhanced BCG elimination *in vivo*. Furthermore, biochemical quantification of M1-M2 polarization in antagonist-treated mice was consistent with the CFU assay results, that is, celecoxib and ONO-AE3-208 suppressed Arg1^+^ M2 polarization in peritoneal BCG-elicited macrophages (Figure S10). These results suggest that A9-dependent PGE2 production significantly affects M1/M2 balance *in vivo*, thereby influencing BCG control.

### Molecular mechanisms of A9-induced Cox-2 upregulation

**Figure 5:**
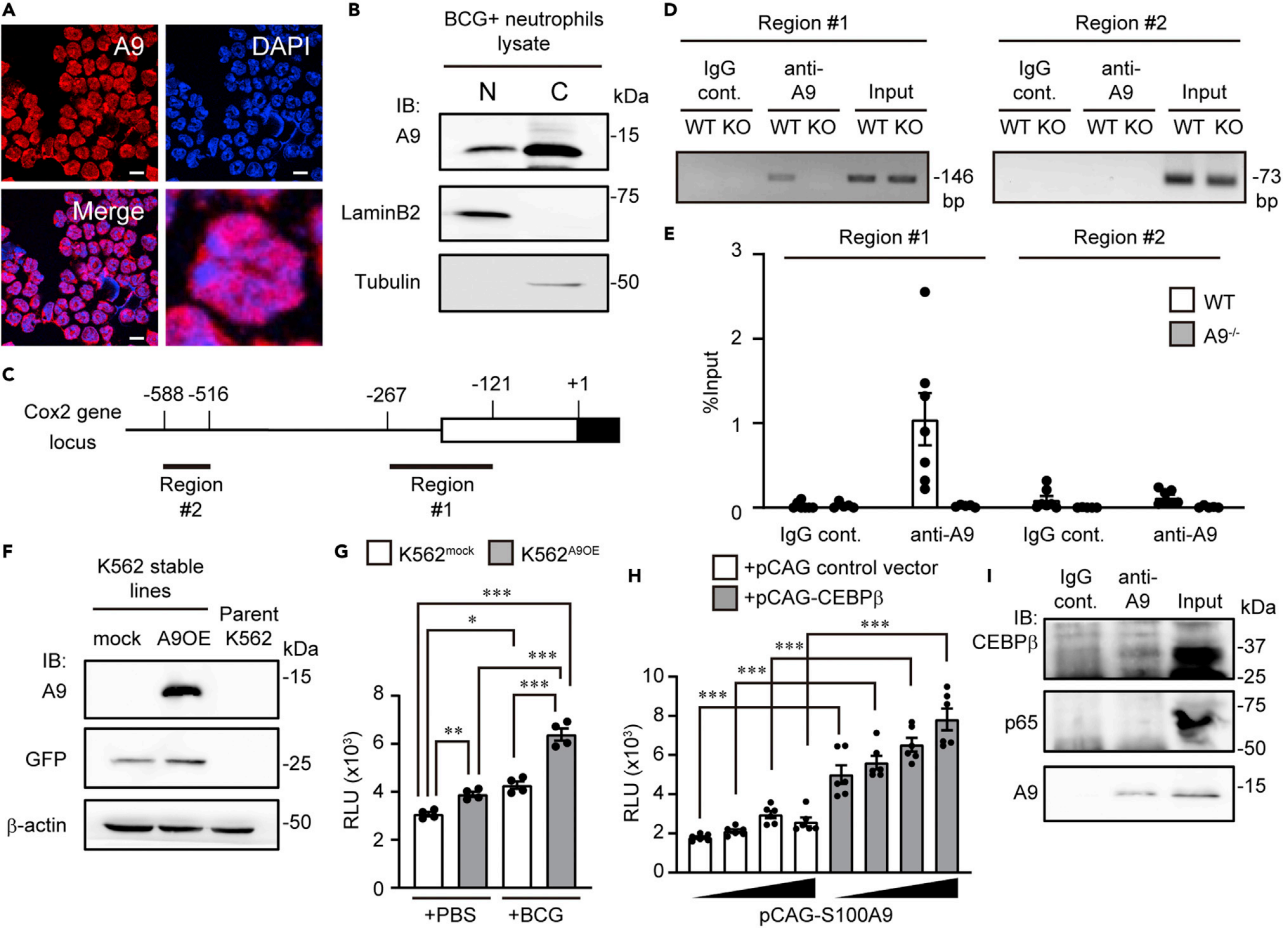
Nuclear A9 interacting with C/EBPβ cooperatively induced *Cox-2* in neutrophils (A) Representative immunofluorescence images of BCG-elicited WT neutrophils with anti-A9 (red) Ab and DAPI (blue) are shown. Scale bars, 10 μm. (B) Immunoblotting using the anti-A9 antibody in the nuclear and cytoplasmic fractions of BCG-elicited neutrophils. Lamin B2 is a nuclear marker, while tubulin is a cytoplasmic fraction marker. (C) Schematic of mouse *Cox-2* gene 5′ regions. The white and black boxes indicate the 5′ UTR and CDS sequences, respectively. Thick black lines below the Cox-2 gene promoter (#1 and #2) indicate the regions amplified by the PCR primer sets for the chromatin immunoprecipitation analysis. (D) Chromatin immunoprecipitation of A9 from purified BCG-elicited neutrophils; regions #1 and #2 in the promoter were detected via PCR analysis with specific primers. (E) ChIP-qPCR assay of the Cox-2 gene promoter in BCG-elicited WT or A9^−/−^ Neu. (F) Stable A9 overexpression cell lines (K562^A9OE^) were constructed. The expression level of A9 was analyzed via immunoblotting. (G) Luciferase reporter activities driven by the Cox-2 promoter were determined in K562^A9OE^ and K562^mock^ cells. After transfection with the luciferase reporter plasmid, pNL-Cox2-luc, and pGL4.54, the cells were simulated with BCG (gray) or PBS (open), and the induction level of luciferase activity was measured. (H) HEK293T cells were transiently co-transfected with pNL-Cox-2-luc, pGL4.54, and the expression vector for the indicated combinations of C/EBPβ and A9 (0, 4, 20, or 100 ng). After 24 h, cells were harvested, and luciferase activity was measured. The mean values of six independent samples are shown. (I) Nuclear A9-associated proteins were examined via co-immunoprecipitation with an anti-A9 antibody and immunoblot with anti-A9, anti-p65, or anti-C/EBPβ antibodies. A9 interacted with C/EBPβ but not with p65 in BCG-stimulated neutrophils. Whole-cell lysate was used as the input. Two biological replicate experiments were performed, and a representative experiment is shown (B, D, and I). Data are from 1 representative experiment (G) or pooled from two independent experiments (E and H) and are shown as mean ± SEM. ∗∗∗p < 0.0005, ∗∗p < 0.005, ∗p < 0.05, ANOVA and post hoc Tukey-Kramer test (E, G, and H).

Apart from its role as an alarmin, A9 has various other functions, including that of a transcriptional co-activator.^35^ Immunofluorescence analysis revealed that part of the A9 fraction was localized within the nucleus of BCG-activated neutrophils (Figure 5A). Nuclear protein Lamin B2 was specifically detected in the nuclear extracts, whereas tubulin was only detected in the cytoplasmic protein fraction (Figure 5B). With such minimal cross-contamination between the cytosolic and nuclear fractions, A9 protein was readily detected in the nuclear fraction (Figure 5B). These results indicate that A9 protein was partially stabilized in the nucleus of neutrophils.

We then determined whether nuclear A9 binds to the Cox-2 gene promoter, as predicted by mouse ENCODE data sets^36^ (Figure 5C). Chromatin immunoprecipitation (ChIP) assay detected A9 binding activity at a transcriptional regulatory region of −267 to −121, but not at the −588 to −516 region of the Cox-2 gene (Figures 5C–5E). Intriguingly, the association between A9 and the Cox-2 promoter was not significantly enriched under casein-induced hypoallergenic conditions, indicating that BCG infection increased A9 binding to the Cox-2 promoters (Figure S11).

We then investigated whether A9 directly activates the Cox-2 gene promoter. To this end, we developed a stable A9-overexpressing K562 cell line, referred to as K562^A9OE^, in addition to a control K562^mock^ line using lentiviral transfer (Figure 5F). BCG stimulation moderately increased Cox-2 promoter activity in both cell lines, with the relative light units (RLUs) of K562^A9OE^ significantly exceeding those of K562^mock^ (Figure 5G). Even in the absence of BCG, we observed a significant difference in the activation of these cell lines, suggesting that abundant A9 expression may activate the Cox-2 promoter in this experimental setting (Figure 5G).

ChIP assays verified that a C/EBPα/β consensus-binding site overlapped with the A9 binding region. C/EBPβ is an essential transcription factor for Cox-2 gene expression. Thus, we examined the cooperative interaction between A9 and C/EBPβ in the transcriptional activity of the Cox-2 promoter. The expression of A9 alone in HEK293T cells did not activate the Cox-2 promoter (Figure 5H); enhanced activation was observed upon C/EBPβ co-expression in a dose-dependent manner.

To assess the physiological interaction between these proteins, we performed a conventional co-immunoprecipitation assay with the nuclear fraction of neutrophils and an anti-A9 antibody. Nuclear A9 was co-immunoprecipitated with C/EBPβ in BCG-activated neutrophils, but was not detected with the nuclear factor κB component p65, a critical transcriptional regulator of the *Cox-2* gene^37^ (Figure 5I). These results support the notion that the DNA-bound A9-C/EBPβ complex strongly upregulates *Cox-2* expression.

### Effect of celecoxib on M2 polarization within granulomas

**Figure 6:**
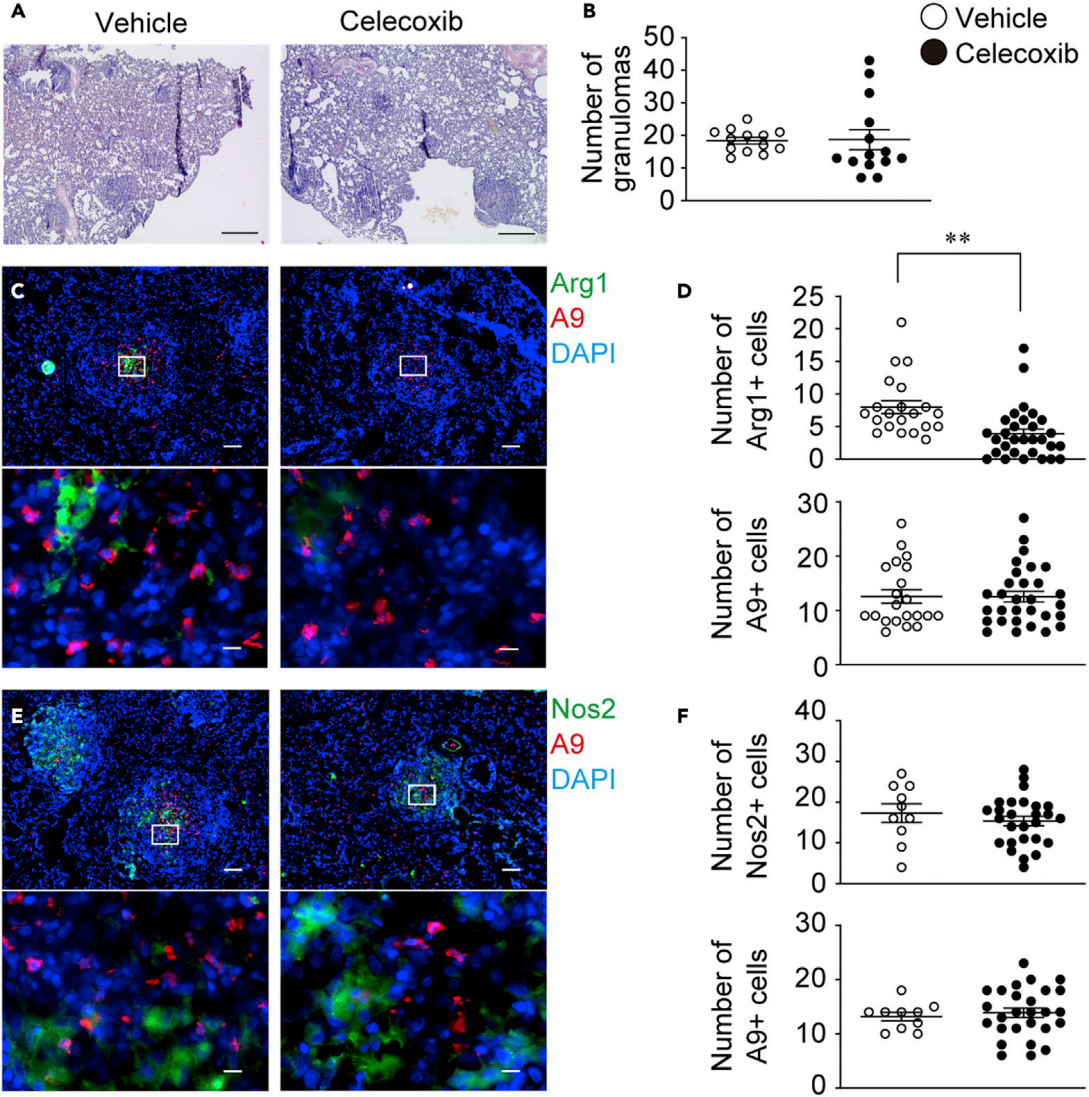
Celecoxib treatment specifically reduced M2 in the granulomas The effect of celecoxib on granulomatous macrophage polarization was evaluated in the guinea pig granuloma model. (A) Lung sections derived from celecoxib-treated (right panel) and vehicle-treated (left panel) guinea pigs were stained with hematoxylin and eosin, with representative micrographs obtained from two independent experiments shown. Scale bars, 500 μm. (B) Granulomas were counted for 3–4 animals per group (vehicle, n = 3; celecoxib, n = 4). The numbers of granulomas per section is shown as dot plots, and the mean values as lines. (C) Lung sections derived from celecoxib-treated and vehicle-treated guinea pigs were labeled with anti-Arg1 (green) and anti-A9 (red) and detected via immunofluorescence staining. Scale bars, 100 μm. The bottom panel shows a magnified view (100×) of the white inset boxes outlined in the top panels. Scale bars, 10 μm. (D) Arg1^+^ and A9^+^ cells in the magnified view of the field were counted for 3–4 animals per group, dot plot data are shown, and the mean values are shown as lines. Vehicle- (○) and celecoxib-treated animals (•). (E) Lung sections derived from celecoxib-treated and vehicle-treated guinea pigs were labeled with anti-Nos2 (green) anti-A9 (red) and detected via immunofluorescence. (F) Nos2^+^ and A9^+^ cells were counted for 3–4 animals per group, dot-plot data are shown, and the mean values are shown as lines. Vehicle- (○) and celecoxib-treated animals (•). Data were pooled from two independent experiments and shown as mean ± SEM. ∗∗p < 0.005, Welch’s *t*-test.

To assess whether COX-2 in neutrophils plays a role in the induction of granulomatous M2 macrophages, celecoxib was administered to BCG-granuloma animals. The overall structure of lung granulomas was similar between celecoxib-treated and vehicle-treated animals (Figure 6A). There was also no significant difference in the number of granulomas in the lungs, albeit with a slight decrease in size under celecoxib treatment when compared to the vehicle control (Figures 6B and S12).

To determine the effect of celecoxib on M2 polarization in granulomas, we randomly extracted magnified images of the center position of granulomas containing abundant A9^+^ cell regions and counted each stained cell (A9, Arg1, and Nos2). The number of A9^+^ neutrophils in the granulomas was comparable between celecoxib-treated and vehicle-treated animals (vehicle: 12.5 ± 1.25 vs. celecoxib: 12.5 ± 0.98, Figures 6C and 6D, vehicle: 13.2 ± 0.786 vs. celecoxib: 13.9 ± 0.881, Figures 6E and 6F). Under a comparable A9^+^ cell count, the number of Arg1^+^ macrophages was significantly lower in celecoxib-treated animals than that in the controls (vehicle: 8.24 ± 0.861 vs. celecoxib: 4.63 ± 0.777, Figures 6C and 6D). The number of Nos2^+^ cells between the groups was not significantly different, in the presence of equivalent cell numbers of A9^+^ neutrophils (vehicle: 17.3 ± 2.28 vs. celecoxib: 15.4 ± 1.15, Figures 6E and 6F). These results support the significant involvement of COX-2 in M2 granuloma development.

## Discussion

The proximal localization between A9^+^ neutrophils and Arg1^+^ M2 macrophages at granulomas prompted us to examine the impact of A9 on M2 induction. We demonstrated that A9^−/−^ mouse neutrophils exhibit an insufficient capacity for M2 induction, and as a result, the A9-dependent induction of Cox-2 expression in neutrophils is pivotal in the development of the M2 region in granulomas.

The granulomas we analyzed in this study were acute inflammatory tissues that developed in BCG-immunized guinea pigs 1 week after the challenge, and their core was filled with neutrophils. In the early stages of *M. tuberculosis* infection, neutrophils are the predominant immune cells that invade tissue and phagocytose mycobacteria,^38^^,^^39^^,^^40^ suggesting that neutrophils accumulating at the center of granulomas recognize massive acute inflammatory lesions. As M2 macrophages are known to serve as a “Trojan horse” for mycobacteria,^14^^,^^41^ A9-driven M2 polarization, although induced at an early phase, may indeed assist bacterial growth, resulting in persistent granulomatous inflammation. In fact, we demonstrated that *in vivo* BCG control at an early phase was enhanced in A9^−/−^ mice compared to that in controls. This notion is supported by a study reporting that *M. tuberculosis* reactivation was markedly reduced in A9^−/−^ mice.^40^ In contrast, human tuberculosis exhibits pro-inflammatory signatures in the center of granulomas,^10^ conflicting with the results of the BCG granuloma analysis in this study. This discrepancy is likely due to the differences in disease time course, host, and bacterial species between experiments. As human pathology specimens are from cases with a severe and recurrent disease course, a significant inflammatory response at the centric granulomas is inferred.

Our observations suggested that A9 deficiency reduces the M2 population compared with WT, thus resulting in a relative increase in the number of bactericidal M1 during BCG infection. This result is of great interest because A9 serum concentration was increased in cases of human inflammatory disorders, in a manner reminiscent of pro-inflammatory cytokines and chemokines.^18^^,^^23^^,^^40^^,^^42^ The A9 forms a heterodimer with A8^18^^,^^19^ and interacts with TLR4 and RAGE, causing downstream pro-inflammatory signaling activation.^23^^,^^43^ Extracellular A9 reportedly assisted M1 polarization for a short duration,^44^ and it has been closely linked to severe inflammation.^45^ Indeed, a significant elevation in the serum level of A9 has been detected in the acute phase of severe lung injury in tuberculosis,^46^ implying that A9 might be transiently assisting M1 inducers in the acute phase. On the contrary, our present study results support that bacteria-elicited neutrophils consistently exerted an M2-inducible effect in an *in vitro* co-culture system and granulomas. Therefore, we speculate that A9 is involved in M2 polarization in a persistent inflamed environment during the acute and latent phases of an infection. Future studies should determine whether A9 switches from an M1-assisting role to that of an M2 inducer through temporal regulation during inflammation. However, A9-dependent M2 polarization may be less noticeable in transient bacterial infections, including *Escherichia coli* infection, and extracellular A9 may be a driving force for bacterial control.^23^ Although our findings suggest that A9-dependent M2 induction occurs in the background of mycobacterial inflammation, this pathway is rather insufficient, especially for the control of mycobacteria, which are capable of feeding on M2. Of note, A9 is essential in repairing arterial injuries^47^ and neutrophilia associated with severe lung damage during progressive *M. tuberculosis* infection.^48^ Thus, the A9-M2 pathway may play an important role in repairing tissue damage caused by bacterial infection. Examination of tissue damage to the lungs during mycobacterial infection in A9^−/−^ mice may, therefore, be of interest.

In this study, nuclear A9 was found to contribute to the transcriptional upregulation of the *Cox-2* gene. However, extracellular alarmin A9 binds a variety of receptors and upregulates the expression of pro-inflammatory genes, including *Cox-2*.^49^ Based on the lesser effect of TasQ on the M1/M2 testing co-culture system, extracellular A9 with alarmin functions is not critical, whereas nuclear A9-mediated *Cox-2* gene transcription is implicated in M2 induction. Thus, Cox-2 induction in neutrophils depends on nuclear A9, at least within 48 h of cell-cell contact. Nevertheless, a general nuclear localization signal is missing for A9. Although A9 may form a complex with nuclear-shuttled proteins, which are transcriptional factors in the cytoplasm, the mechanism of A9 translocalization into the nucleus remains unclear and should be further explored. Owing to the complex molecular dynamics of A9, A9-dependent Cox-2 transcription might involve several regulatory steps with intracellular and extracellular A9 dynamics. Thus, the localization and function of A9 in relation to Cox-2 expression in neutrophils require further investigation.

The observed physical interactions between neutrophils and macrophages may occur in other conditions, such as malignant tumors. Neutrophils are frontline immune cells that eliminate bacteria through reactive oxygen species and neutrophil extracellular traps.^50^ Similar to an anti-bacterial response, tumor-associated neutrophils (TANs) can eliminate cancer cells through these effector mechanisms.^51^ Recent insights into neutrophil phenotypes have revealed the existence of anti-inflammatory neutrophils called N2-TAN or granulocytic myeloid-derived suppressor cells (gMDSCs), which support tumor progression.^52^ Histological observation of human cancers revealed M2 enrichment characterized by the presence of gMDSCs, suggesting physical interactions between gMDSCs and M2-polarized macrophages in several cancer types.^53^^,^^54^ Importantly, Cox-2 expression is an essential event in the emergence of high A9-expressing MDSCs.^55^ Therefore, the A9-Cox-2 pathway identified herein may be involved in the emergence of M2 macrophages and gMDSCs within the tumor microenvironment. Given that COX-2-PGE2 signaling is governed by neutrophil A9 during tumor progression, the manipulation of A9 translocation from the cytoplasm to the nucleus may open new avenues for cancer treatments.

### Limitations of the study

This study had a few limitations. We validated the contribution of the A9-COX-2 axis to M2 polarization in the granulomas using the selective COX-2 inhibitor celecoxib. However, *in vivo* celecoxib treatment may have influenced other Cox-2^+^ cells, in addition to neutrophils. Eosinophils have been detected in tuberculosis granulomas, albeit at a low level,^56^ and they utilize COX-2 signaling to produce M2 cytokines.^57^ Moreover, during bacterial infection, celecoxib can alter the gut microbiota composition, which is followed by a decrease in lung M2 macrophage proportion.^58^ Future research should focus on the contribution of neutrophil A9-Cox2 signaling to the formation of the granuloma M2 niche. For this purpose, studies should use animals with conditional A9 or Cox-2 deletion in neutrophils or use novel A9 inhibitors that specifically inhibit the functions of not only the chemoattractant but also cytoplasmic A9 during lung granuloma formation. Second, nuclear A9 could also interact with CEBP/β to promote Cox-2 upregulation. Although the A9-bound DNA region was identified in the Cox-2 promoter, we did not explore the structure and specificities of the DNA sequence to which nuclear A9 can bind. In addition, we did not address the spatiotemporal regulation of the nuclear translocalization of A9. The assessment of genome-wide chromatin accessibility of A9 will help elucidate the molecular mechanism underlying the DNA-binding activity of A9.

## STAR★Methods

### Key resources table

**Table:** 

REAGENT or RESOURCE	SOURCE	IDENTIFIER
**Antibodies**		

Rat monoclonal anti-guinea pig S100A9	Yoshioka et al.^16^	N/A
Rat monoclonal anti-guinea pig Arginase1	This study	N/A
Rabbit polyclonal anti-guinea pig Nos2	This study	N/A
Rabbit monoclonal anti-Arginase1 (D4E3M)	Cell Signaling Technology	Cat#936685
Rabbit monoclonal anti-Nos2 (D6B6S)	Cell Signaling Technology	Cat#131205
Rabbit polyclonal anti-β-actin	Cell Signaling Technology	Cat#49675
Rabbit anti-S100A9 (D3U8M)	Cell Signaling Technology	Cat#73425; AB_2799839
Rabbit polyclonal anti-LaminB2	Proteintech	Cat#10895-1-AP; AB_2136411
Rabbit polyclonal anti-Tubulin	Cell Signaling Technology	Cat#2148; AB_2288042
Rabbit polyclonal anti-C/EBPβ	Cell Signaling Technology	Cat#3087; AB_2078052
Rabbit polyclonal anti-NF-kB p65	Cell Signaling Technology	Cat#8242; AB_10859369
Rat APC anti-mouse F4/80 (BM8)	BioLegend	Cat#123116; AB_893481
Rat FITC anti-mouse CD11c (HL3)	BD Biosciences	Cat#553801; AB_396683
Brilliant Violet 421 anti-mouse CD11b (M1/70)	BioLegend	Cat#101235; AB_10897942
PE anti-mouse Ly6G (1A8)	BioLegend	Cat#127608; AB_1186099
Alexa Fluor 647 anti-CD206 (C068C2)	BioLegend	Cat#141712; AB_10900420
Alexa Fluor 488 anti-CD68 (FA-11)	BioLegend	Cat#137012; AB_2074846
Alexa Fluor 647 anti-mouse S100A9 (2B10)	BD Biosciences	Cat#565833; AB_2739373
Purified Rabbit Polyclonal Isotype Ctrl	BioLegend	Cat#910801; AB_2722735
HRP-conjugated goat anti-rabbit IgG	Jackson ImmunoResearch Lab	Cat#110-035-1442; AB_2307391
Purified Rat Anti-Mouse CD16/CD32	BD Biosciences	Cat#553142; AB_394656
Alexa Fluor 647 Rat IgG2a, κ Isotype Ctrl	BioLegend	Cat#400526; AB_2864284
Alexa Fluor 488 Rat IgG2a, κ Isotype Ctrl	BioLegend	Cat#400525; AB_2864283
Alexa Fluor 647 Rat IgG1, κ Isotype Ctrl	BioLegend	Cat#400418; AB_389341

**Bacterial and virus strains**		

*Mycobacterium bovis bacillus Calmette-Guerin*: strain Tokyo 172	N/A	N/A

**Chemicals, peptides, and recombinant proteins**		

Tasquinimod	MedChem Express	HY-10528
Human M-CSF	Peprotech	Cat#300-25
Celecoxib	Sigma-Aldrich	Cat#PHR1683
ONO-AE3-208	ChemScene	Cat#CS-0315

**Critical commercial assays**		

Vectastain ABC standard kit	Vector Labs	Cat#1PK-4000
Alexa Fluor 488 Protein Labeling Kit	Invitrogen	Cat#A10235
Alexa Fluor 594 Protein Labeling Kit	Invitrogen	Cat#A10239
Mouse neutrophil isolation MicroBeads	Miltenyi Biotec	Cat#130-097-658
ReverTra Ace qPCR RT master kit	TOYOBO	Cat#FSQ-201
PowerUp SYBR Green master Mix	Thermo Fisher	Cat#A25742
RNeasy Plus Mini Kit	QIAGEN	Cat#74136
PGE2 Assay kit	R&D systems	Cat#KGE004B
Casein (7.5%) Cayman Chemicals	Cayman Chemicals	Cat#601070
LTB4 ELISA Kit	Cayman Chemicals	Cat#520111
EZ-Magna ChIP® G-Chromatin Immunoprecipitation Kit	Millipore	Cat#17-409
Nano-Glo® Dual-Luciferase® Reporter Assay System	Promega	Cat#N1610

**Deposited data**		

RNA-seq	This paper	GEO: GSE162991

**Experimental models: Organisms/strains**		

Guinea pig: Slc:Hartley	Japan SLC	N/A
Mouse: S100A9^−/−^	This study	N/A
Mouse: C57/BL6JJmsSlc	Japan SLC	N/A

**Experimental models: Cell lines**		

Human: K562	ATCC	N/A
Human: K562-A9OE	This study	N/A
Human: K562-mock	This study	N/A
Human: HEK293T	ATCC	N/A

**Oligonucleotides**		

qPCR primers	This paper	See Table S1
ChIP-qPCR primers	This paper	See Table S1
Primer: Mouse S100A9 gene sequencing (Fwd): TAGGTCAGGGAAGCTTGGTTATC	This paper	N/A
Primer: Mouse S100A9 gene sequencing (Rev): TGTTCAACTGAACCATTTCCTGA	This paper	N/A

**Recombinant DNA**		

Plasmid: pX330-S100a9-gRNA	This paper	N/A
Plasmid: pX330-U6-Chimeric_BB-CBh-hSpCas9	Addgene	Cat#42230
Plasmid: pCSII-CMV-MCS-IRES2-Venus	RIKEN BRC	RDB04383
Plasmid: pCSII-CMV-S100A9-IRES2-Venus	This paper	N/A
Plasmid: pCAG-HIVgp	RIKEN BRC	RDB04394
Plasmid: pCAG-VSV-G-RSV-Rev	RIKEN BRC	RDB04393
Plasmid: pCAG-FLAG-S100A9	This paper	N/A
Plasmid: pNL1.1	Promega	Cat#N1001
Plasmid: pGL4.54	Promega	Cat#E5061
Plasmid: pCAG-C/EBPβ	This paper	N/A
Plasmid: pNL-Cox2	This paper	N/A

**Software and algorithms**		

CHOPCHOP v2	Labun et al.^59^	http://chopchop.cbu.uib.no/
ImageJ	US National Institutes of Health	N/A
FlowJo V10	TreeStar	N/A
HISAT2 (ver. 2.1.0)	Kim et al.^60^	http://daehwankimlab.github.io/hisat2/
DEseq2 (ver. 1.26.0)	Love et al.^61^	https://bioconductor.org/packages/release/bioc/html/DESeq2.html
Database for Annotation, Visualization, and Integrated Discovery (DAVID)	Huang et al.^31^	https://david.ncifcrf.gov/
Reactome	Jassal et al.^62^	https://reactome.org/
PRISM software (version 9.4.1)	GraphPad	N/A

**Other**		

NovaSeq 6000 system	Illumina Inc	N/A
LAS-4000mini	GE Healthcare	N/A
ABI7500 system	Applied Biosystems	N/A
ARVO-X3	PerkinElmer	N/A
BZ-X710	Keyence	N/A
FV1000	Olympus	N/A
FACS-Aria III	Becton Dickinson	N/A
LSRFortessa X-20	Becton Dickinson	N/A

### Resource availability

#### Lead contact

Further information and requests for resources should be directed to and will be fulfilled by the lead contact, Tatsuaki Mizutani (mizutani@infront.kyoto-u.ac.jp).

#### Materials availability

Materials generated in this study can be made available upon request to the lead contact.

### Experimental models and subject details

#### Animals

Animal experiments were performed in accordance with institutional guidelines on animal welfare and were approved by the Kyoto University Animal Experimentation Committee. Three-week-old female Hartley guinea pigs and eight- to ten-week-old female and male C57/BL6*J* mice were purchased from Japan SLC, Inc. (Shizuoka, Japan), and housed under specific pathogen-free conditions.

#### Culture and quantification of BCG

BCG (strain Tokyo 172) was cultured in Middlebrook 7H9 medium enriched with oleic acid-albumin-dextrose-catalase supplement (BD Biosciences, Heidelberg, Germany) until the mid-log phase and then frozen in 1-mL aliquots at −80°C until use. To quantify CFU, mycobacteria were serially diluted in Middlebrook 7H9 medium and plated on Middlebrook 7H10 agar plates (BD Biosciences). After three weeks of incubation at 37°C, the CFU was quantified.

#### Guinea pig granuloma model

Guinea pigs received an intradermal injection of the vaccine strain BCG (1 × 10^8^ CFU per animal) for sensitization. After six weeks, an intravenous injection of BCG (1 × 10^8^ CFU per animal) was administered to induce the formation of granulomas in the lungs.^16^

#### Generation of *S100a9*-deficient mice

We generated *S100a9*-deficient mice using the CRISPR/Cas9 system. To select Cas9 target sites in the *S100a9* gene, we used the CRISPR design tool (http://chopchop.cbu.uib.no/) to minimize off-targeting effects. We subsequently chose a target site, corresponding to exon 2 coding for the N-terminal region (5’ -GAA GGA ATT CAG ACA AAT GG-3′). The gRNA oligonucleotides were inserted into the BbsI site of pX330-U6-Chimeric_BB-CBh-hSpCas9 (Addgene, Cambridge, MA) to generate pX330-S100a9 gRNA. The plasmid was purified using the plasmid midi kit (Qiagen, Chatsworth, CA), and then injected into the pronuclei of fertilized eggs to generate mutant mice.

After isolation of genomic DNA from F0 mutant mice, exon 2 of the *S100a9* gene, including the gRNA target site, was amplified by PCR using KOD FX DNA polymerase (Toyobo, Osaka, Japan) with forward (5’ -CTT CCT CTT GAA GCC CTC CT-3′) and reverse primers (5′-TCT CAC CAT CCT CCC AAC TC-3′), subcloned into pCRII-Blunt-Topo vector (Invitrogen Life Technologies, Gaithersburg, MD), and sequenced. The sequencing reactions were performed using the Big Dye Terminator v3.1 Cycle Sequencing kit (Applied Biosystems, Foster City, CA) according to the manufacturer’s recommendations. The sequencing products were analyzed on an ABI 3130xl Genetic Analyzer (Applied Biosystems) and Genetyx (Genetyx Corporation, Tokyo, Japan).

Four pups from the Cas9/sgRNA expressing plasmid DNA termed pX330-S100a9-gRNA injection were founders bearing four different mutations at the exon 2, as determined by DNA sequencing. The longest deletion was observed in one founder, with a 50 bp deletion. This most considerable mutation was transmitted efficiently to the next generation; however, offspring mice developed normally, and no overt differences were observed in hematopoietic cell populations in the bone marrow and spleen.

### Method details

#### Immunohistochemistry

Chemical reagents were purchased from Nacalai Tesque (Kyoto, Japan) unless otherwise indicated. Immunohistochemistry of guinea pig lung granulomas was performed as previously described.^16^ In brief, isolated lungs were fixed with 4% paraformaldehyde (PFA) and deep-frozen in OCT compound (Sakura Finetechnical, Tokyo, Japan). Cryosections were pre-treated with 3% hydrogen peroxide and 5% guinea pig serum and incubated with an anti-Arg1 antibody (Ab). Labeled cryosections were then incubated with biotinylated secondary antibodies (Dako, Carpinteria, CA), followed by chromogenic reactions with 3,3′-diaminobenzidine (DAB) using the ABC horseradish peroxidase (HRP) kit (Vector Laboratories, Burlingame, CA). Tissue samples were counterstained with hematoxylin (Wako chemicals, Osaka, Japan).

For immunofluorescence imaging, cryosections were treated with 5% guinea pig serum for 30 min, followed by incubation with the following fluorescence-labeled lab-made Abs: anti-S100A9, anti-Nos2, and anti-Arg1 Abs. Isotype-matched Abs (BioLegend, San Diego, CA) were used as the negative control in each experiment. Stained sections were viewed under the Keyence BZ-X710 microscope (Keyence, Osaka, Japan). To evaluate the effect of celecoxib on M1-M2 polarization in the granuloma, two comparable sections were prepared from the left upper lobe of the lung of each animal, and after the labeling with the antibodies as described above, and the number of granulomas in a lung section and that of S100A9^+^, Arg1^+^ or Nos2^+^ cells in granulomas (100×) were counted.

#### Co-culture system of mice peritoneal neutrophils with macrophages

Mice were injected *i.p.* with BCG (5 × 10^7^ CFU) or 7.5% casein solution (Cayman Chemical, Ann Arbor, MI) in 500 μL PBS. After 6 h, peritoneal exudate cells (PECs) were collected from peritoneal lavage with 10 mL PBS, and peritoneal neutrophils were immediately isolated using a MACS column with neutrophil isolation MicroBeads (Miltenyi Biotec, Sunnyvale, CA). Mouse WT or A9^−/−^ BM cells were isolated by flushing femurs and tibias with 5 mL PBS supplemented with 2% heat-inactivated fetal bovine serum (FBS; HyClone, Logan, UT). The BM cells were centrifuged once and then suspended in RBC Lysis buffer (BioLegend) at room temperature (22–24 °C) for 3 minutes to lyse red blood cells. The cells were centrifuged again and then suspended in Dulbecco’s modified Eagle’s medium (DMEM; Invitrogen Life Technologies) supplemented with 10% FBS, and cultured at 1 × 10^6^ cells/mL with 10 ng/mL human M-CSF (Peprotech) in petri dishes (diameter 90 mm, Sansei Medical., Kyoto, Japan). After 6–7 days of culture, non-adherent cells were removed, and the adherent macrophages were collected and used for co-culture experiments with neutrophils. The peritoneal neutrophils from WT or A9^−/−^ mice were added at a 1:1 ratio to the BMMs. Cells were cultured in DMEM with 10% FBS, 2 mM glutamine, 100 U/mL penicillin, and 100 μg/mL streptomycin at 37°C in the presence of 5% CO_2_. After co-culture for 48 h, RNA was extracted from the adherent macrophages and analyzed, or cells were fixed and used for immunofluorescence analysis. In select experiment, cells were incubated in the presence of DMSO or TasQ (5 μM) and harvested at the indicated time points. For the blockade of COX-2 in neutrophils, before the co-culture, neutrophils were treated with celecoxib (1 μM, Sigma-Aldrich, St Louis, MO) for 15 min at 37°C. For prostaglandin-E2 (PGE2) receptors (EP) antagonist treatment, 1 μM ONO-AE3-208 (ChemScene, LLC, Newark, NJ), or AH6809 (Cayman Chemical) or L-798,106 (Cayman Chemical) was added to the BMMs for 1 h at 37°C. After washing with the medium, the neutrophils were added to the BMMs culture wells.

#### Immunofluorescence

Before the fixation, non- and semi-adherent cells were removed by PBS wash, and the adherent cells as macrophages were used in immunofluorescence analysis. Cells were fixed with 4% PFA at room temperature (22–24 °C) for 30 min, permeabilized with 0.5% saponin/2% FBS/PBS or 0.5% saponin/5% FBS/PBS at room temperature for 30 min, then incubated with Alexa Fluor 488 anti-CD68 (FA-11, BioLegend) and Alexa Fluor 647 anti-CD206 Abs at room temperature for 30 min. Isotype-matched Ab (BioLegend) was used as the negative control in each experiment. The BD Perm/Wash™ buffer (BD Biosciences) was used throughout the incubation with the antibodies and for all washes. The labeled cells were mounted in Prolong-Gold™ with DAPI (Invitrogen Life Technologies), and viewed under a fluorescence microscope Keyence BZ-X710 (Keyence). Images were acquired using a Keyence Imaging Scope (Keyence) at 40× and 100× magnification. A minimum of 10 fields were acquired, and CD68^+^ DAPI^+^ or CD206^+^ DAPI^+^ cells were counted using the Hybrid Cell Count software (BZ-II Analyzer; Keyence). For intracellular S100A9 labeling in murine peritoneal neutrophils, cells were fixed and permeabilized with the Fixation/Permeabilization solution (BD Biosciences) and labeled by incubation with fluorescence-conjugated indicated monoclonal antibodies as follows: Alexa Fluor647 anti-mouse S100A9 Ab (clone: 2B10, BD Biosciences). The labeled cells were visualized under a confocal laser scanning microscopy FV1000 (Olympus, Tokyo, Japan) with a 100× oil immersion objective lens.

#### Quantitative RT-PCR

Cells were harvested by a standard procedure and total RNA was extracted using the RNeasy mini kit (Qiagen). Total RNA was reverse-transcribed using ReverTra Ace qPCR RT Master Mix (Toyobo). The resulting cDNA was then subjected to real-time PCR analysis with PowerUp SYBR Master Mix (Thermo Fisher Scientific, San Jose, CA) on the ABI7500 system (Applied Biosystems). Relative gene expression levels were obtained by normalizing target gene cycle threshold (CT) values to the CT values of the *β2-microglobulin* gene. The sequences of the sense and antisense primers are listed separately (Table S3).

#### Immunoblotting

BCG-elicited PECs were collected at 6 days post-BCG injection and seeded in 12-well plate to macrophage adhesion for 3 h. Non-adherent cells were removed by PBS wash, and the adherent cells were used as macrophages. Macrophages were lysed with 0.5% TritonX-100, 50 mM Tris-HCl, pH 7.5, 150 mM NaCl, and a protease inhibitor cocktail (Sigma-Aldrich). Following the removal of insoluble material by centrifugation, cellular proteins were resolved on 8-10% SDS-PAGE gels and transferred onto polyvinylidene difluoride membranes (Bio-Rad, Hercules, CA). After blocking with 5% BSA (Sigma-Aldrich) in 0.05% Tween 80/PBS, the membranes were incubated with either anti-Arg1 (D4E3M, Cell Signaling Technology, Beverly, MA) or anti-Nos2 (D6B6S, Cell Signaling Technology) Abs, followed by HRP-conjugated anti-rabbit IgG Abs (Jackson ImmunoResearch Laboratories). Luminol-based detection was conducted with the ECL Western Blotting Detection Reagent (GE Healthcare, Little Chalfont, UK) according to the manufacturer’s instructions, and signals were analyzed using the LAS-4000mini image analyzer (GE Healthcare). To detect β-actin, all of the attached reagents were removed from the membranes and re-probed with polyclonal Abs against β-actin (Cell Signaling Technology), followed by HRP-conjugated anti-rabbit IgG Abs. Blot intensity was quantified using ImageJ software (US National Institutes of Health). Nuclear and cytoplasmic cell extracts from BCG-elicited neutrophils (5 × 10^6^) were prepared using the NE-PER nuclear and cytoplasmic extraction reagents (Thermo Fisher Scientific), respectively, according to the manufacturer’s recommendations. The fractionated cell extracts were resolved on 10–14% SDS-PAGE gels and subjected to the immunoblot assay using the following antibodies; anti-S100A9 (Cell Signaling Technology, #73425), anti-LaminB2 (ProteinTech, #10895-1-AP), and anti-Tubulin (Cell Signaling Technology, #2148) Abs.

#### BCG colony-forming unit (CFU) assay

In the CFU assay of mouse *i.p.* BCG infection model, peritoneal cells were collected by centrifugation of the abdominal lavage fluid at 1 week after BCG administration. The collected peritoneal cells (1 × 10^5^) were lysed with 1% Triton X-/PBS and then spread on 7H10 plates. To quantify the CFU, duplicative or triplicated samples were serially diluted in Middlebrook 7H9 medium and plated on Middlebrook 7H10 agar plates, CFU was counted after 3–4 weeks of incubation at 37°C.

#### RNA-seq

After 6 h of BCG or casein *i.p.* injections, WT and A9^−/−^ peritoneal neutrophils were collected using the mouse neutrophils isolation MACS kit as described above, and total RNA was isolated using the RNeasy mini kit (Qiagen). Whole transcriptome analysis was performed by RNA-seq on the NovaSeq 6000 system (Illumina Inc., San Diego, CA) by Macrogen Japan (Tokyo, Japan). Raw data were mapped to the mouse genome mm10/GRCm38 using HISAT2 (ver. 2.1.0) and analyzed for DEGs using DEseq2 (ver. 1.26.0). Significant DEGs were selected with adjusted p value (FDR) < 0.05. Pathway analysis was performed using the following bioinformatics resources; Database for Annotation, Visualization, and Integrated Discovery (DAVID, https://david.ncifcrf.gov/) and Reactome (https://reactome.org/). The RNA-seq data generated in this study are available for download from the NCBI Sequence Read Archive under the accession number GSE162991.

#### Enzyme-linked immuno-sorbent assay (ELISA)

To measure secreted PGE2, leukotriene (LT) B4 and TNFα, BCG-elicited WT, and A9^−/−^ neutrophils were incubated in the DMEM medium for 18 h before harvesting of culture supernatant for the assay. The levels of PGE2 and LTB4 present in the culture supernatants were measured using the PGE2 Assay kit (R&D systems, Minneapolis, MN) and the LTB4 ELISA Kit (Cayman Chemicals), respectively, following the manufacturer’s protocol. Plates were read at an optical density of 405 nm on an ARVO-X3 microplate reader (PerkinElmer, Waltham, MA).

#### *In vivo* treatment with COX-2 inhibitor and EP antagonist

Six hours before the intravenous injection of BCG, the Hartley guinea pigs received an *i.p.* injection of celecoxib (50 mg per kg body weight) in dimethyl sulfoxide (DMSO) diluted with saline, sonicated and then vortexed, followed by additional celecoxib injections every 2 days throughout the granuloma induction period. DMSO (10%) was used as the control vehicle. In some experiments, immediately after BCG *i.p*. injection, mice were divided into EP4-antagonist ONO-AE3-208 and vehicle-treated groups, with 3–4 mice per group. ONO-AE3-208 was dissolved in ethanol, and diluted with sterile water. Mice were administered drinking water with vehicle (0.5% ethanol) or ONO-AE3-208 (50 μg/mL) for 1 week starting from the day of BCG injection. In mice with celecoxib treatment, 6 h before BCG infection, they were administered celecoxib at concentrations of 50 mg/kg or vehicles and injected intraperitoneally once a day with celecoxib or with vehicles only.

#### Chromatin immunoprecipitation assay (ChIP)

ChIP assay was carried out using the Magna ChIP Assay Kit (Millipore, Bedford, MA). Briefly, mouse peritoneal neutrophils (5 × 10^6^) were fixed with 1% formaldehyde, washed with ice-cold PBS, and resuspended in SDS lysis buffer. The suspensions were sonicated using an Astrason XL2020 ultrasonic disrupter (Misonix, Farmingdale, NY) to yield 200–600 bp genomic DNA fragments. After centrifugation, supernatants were diluted with ChIP dilution buffer. Then, the samples were incubated with anti-S100A9 Ab (Cell Signaling Technology) or control rabbit IgG (Biolegend) overnight at 4°C. Following incubation with 25 μL of 50% magnetic protein G beads, the samples were washed twice, each with low-salt buffer, high-salt buffer, LiCl buffer, and TE buffer. The immunoprecipitated chromatin complex was eluted and incubated at 65°C overnight to reverse the cross-linking. After proteinase K treatment, DNA was purified by spin column and resuspended in TE buffer. After sonication, aliquots of equal volumes from the samples were used as input controls. The PCR primers (listed in Table S3) were designed to amplify the proximal promoter regions of mouse Cox-2 (−121, −267, or −516, −588). The DNA positions are denoted relative to the transcriptional start site (+1).

#### Plasmid construction

The mouse *S100a9* gene was amplified by PCR from the cDNA of mouse bone marrow cells using the oligonucleotide primers as indicated in Table S3. The resulting PCR DNA fragment was digested with the restriction enzymes XhoI and NotI, and inserted into the pCAG-FLAG plasmid (a gift from Tadatsugu Taniguchi, Tokyo University), resulting in pCAG-FLAG-A9. Similarly, the pCSII-A9-IRES-Venus vector was constructed. Briefly, the S100A9 gene was amplified by PCR as described above, digested with the restriction enzymes XhoI and NotI and inserted into the pCSII-CMV-IRES-Venus plasmid (RIKEN, Yokohama, Japan). To construct mouse C/EBPβ expression vectors, the CDS regions of the C/EBPβ gene were amplified using cDNA extracted from mouse bone marrow cells using specific primers (Table S3) and inserted into the pCAG-Neo PA tag-C vector (FUJIFILM Wako Pure Chemical Corporation, Osaka, Japan). The *Cox-2* gene promoter from −600 + 1 bp was inserted between the KpnI and XhoI sites into the pNL1.1 reporter vector upstream of the NanoLuc luciferase gene (Promega, Madison, WI). The DNA of the Cox-2 promoter region was generated by Eurofins Genomics Inc (Tokyo, Japan). All plasmid DNAs were prepared using a miniprep or midiprep DNA isolation kit (Qiagen).

#### Recombinant lentivirus production

Lentiviral vector and packaging plasmids, pCSII-CMV-MCS-IRES-Venus, pCAG-HIVgp, and pCAG-VSV-G-RSV-Rev plasmids, were obtained from RIKEN BioResource Center (Tsukuba, Japan).^63^^,^^64^ HEK293T cells in 6-well plates were transfected with 1.2 μg of pVSVg, 1.2 μg of psPAX2, and 2 μg of pCSII-A9-IRES-Venus or pCSII-IRES-Venus (mock) vectors by using Lipofectamine 3000 according to the manufacturer’s instructions (Invitrogen Life Technologies). The culture supernatant containing viral vector particles was harvested 48 h after transfection, filtered with a 0.45-μm membrane filter (Millipore), and concentrated using a Lenti-X concentrator (Takara, Shiga, Japan) according to the manufacturer’s protocol. Titers were determined by infection of HEK293T cells. The Venus expression of transduced cells was analyzed on a FACS-LSR Fortessa (BD Biosciences). In routine preparations, the virus titer was ∼10^7^ transduction units/mL. HEK293T cells were cultured in Dulbecco’s modified Eagle’s medium (DMEM) supplemented with 10% fetal calf serum and 1% penicillin-streptomycin under humidity and 10% CO_2_ at 37°C.

#### Generation of stable cell lines

K562 cells were infected by incubation with a concentrated lentiviral vector overnight in the presence of 6 μg/mL polybrene. Four to five days after the infection, cells were analyzed by flow cytometry, and Venus^+^ cells were sorted with a FACS-AriaIII (BD Biosciences) to generate an A9-expressing or control cell line named K562^A9OE^ or K562^mock^, respectively. The K562 cells were grown in RPMI-1640 supplemented with 4 mM L-glutamine (Invitrogen Life Technologies), 10% FBS, and 1% penicillin-streptomycin under humidity and 10% CO_2_ at 37°C.

#### Luciferase assay

K562^mock^ or K562^A9OE^ cells (1 × 10^6^ cells per well) were cultured on a 12-well plate, and 1 μg of reporter plasmid (pNL-mCox-2-luc) was transfected with 0.1 μg of the firefly luciferase control vector pGL4.54 (Promega) with Lipofectamine-LTX reagent (Invitrogen Life Technologies). After transfection for 24 h, cells were divided into BCG and saline-treated groups, and cells were harvested 24 h after BCG stimulation. In some experiments, 293T cells (1×10^5^) were cultured for one day and transfected with 100 ng of pNL-mCox-2-luc, 5 ng of the pGL4.54 (Promega), and 2.5, 25, or 250 ng of the mouse A9 expression vector with 100 ng of pCAG-C/EBPβ or empty control vectors. After transfection for 20 h, the cells were harvested. Cell lysates were assayed for luciferase activity using the Nano-Glo Dual-Luciferase Assay System (Promega) according to the manufacturer’s instructions. Chemiluminescence was measured using the ARVO-X3 system (PerkinElmer, Waltham, MA), and the promoter activities were calculated by the ratio of the respective AU values of Nluc/Fluc. pNL1.1, an empty vector, was used as a negative control.

#### Co-immunoprecipitation

The neutrophil nuclear extracts were incubated with 20 μL of anti-S100A9 (Cell Signaling Technology) or control rabbit IgG antibodies (Abcam, Cambridge, UK) overnight at 4°C. The sample (500 μL) was incubated with 50 μL of protein G-Sepharose beads (GE Healthcare). After washing thrice with ChIP dilution buffer, the beads were eluted with 20-50 μL of Laemmli buffer. After completion of the immunoprecipitation, equal sample volumes were analyzed by immunoblot, as described above using the following antibodies: anti-C/EBPβ (#3087; Cell Signaling Technology), anti-p65 (Cell Signaling Technology), and anti-S100A9 antibodies.

#### Flow cytometry

Cells were plated in 96-well Flat-bottom plates (BD Biosciences) and preincubated with 1 μg Fc Block (BD Biosciences) per 106 cells in 100 μL of PBS containing 2% FBS and 0.02% sodium azide (FACS staining buffer) for 15 min at 4 °C. The cells were stained with the following fluorescent Abs which are specific for cell surface antigens as follows: APC anti-mouse F4/80 (clone: BM8, BioLegend), FITC anti-mouse CD11c (HL3, BD Biosciences), Brilliant Violet (Bv) 421 anti-mouse CD11b (M1/70, BioLegend), and PE anti-mouse Ly6G (1A8, BioLegend) Abs. The cells were washed three times with the FACS staining buffer, acquired on a BD LSRFortessa flow cytometer (BD Biosciences), and analyzed using FlowJo software (Tree Star Inc., Ashland, OR). Dead cells were identified by propidium iodide (PI) staining (BD Biosciences).

### Quantification and statistical analysis

Statistical significance was set at ∗p < 0.05, ∗∗p < 0.005∗ and ∗∗∗p < 0.0005. Simple linear regression and all statistical analyses were performed in GraphPad Prism software (version 9.4.1; GraphPad Software LLC, San Diego, CA). Two-tailed Welch’s *t*-test was used to analyze differences between the means of the two groups. Multiple groups were compared by analysis of variance (ANOVA) followed by Tukey−Kremer’s multiple comparison test. Sample sizes are indicated in each corresponding figure legend.

## Data Availability

• This paper does not report original code. • RNA sequencing data were deposited in NCBI’s Gene Expression Omnibus and are publicly available as of the date of publication. Accession numbers are listed in the key resources table. Any additional information required to reanalyze the data reported in this paper is available from the lead contact upon request.

## Supplemental information

Document S1. Figures S1–S12 and Table S3

Table S1. Functional pathway enriched in the list of DEGs (BCG-elicited WT vs. S100A9^−/−^ neutrophils), related to Figure 4 The 10 most significant clusters are shown (p < 0.05).

Table S2. Functional pathway enriched in the list of DEGs (casein-elicited WT vs. S100A9^−/−^ neutrophils), related to Figure 4 The 10 most significant clusters are shown.
