# Supplementary material for: Neutrophil S100A9 supports M2 macrophage niche formation in granulomas
Source: iScience. 2023 Jan 31;26(3):106081. doi: 10.1016/j.isci.2023.106081 (PMC9947307; doi:10.1016/j.isci.2023.106081)
Supplement: Document S1. Figures S1–S12 and Table S3 [file mmc1.pdf]

iScience, Volume *26*

Supplemental information

Neutrophil S100A9 supports M2 macrophage niche formation in granulomas

Tatsuaki Mizutani, Toshiaki Ano, Yuya Yoshioka, Satoshi Mizuta, Keiko Takemoto, Yuki Ouchi, Daisuke Morita, Satsuki Kitano, Hitoshi Miyachi, Tatsuaki Tsuruyama, Nagatoshi Fujiwara, and Masahiko Sugita

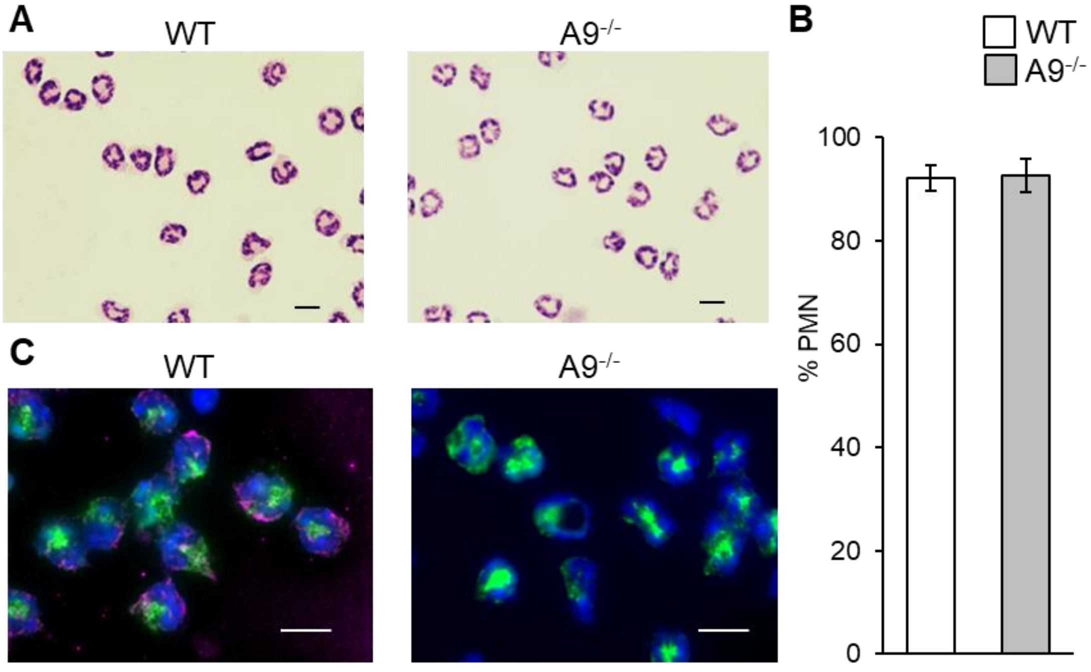

Figure S1. Cytospin analyses of purified mouse peritoneal neutrophils, related to Fig. 2. BCG-elicited mouse peritoneal exudate cells sorted with MACS as described in STAR Methods. (A) Purified neutrophils display a typical morphology with multi-lobulated nuclei on cytospin preparation after May–Grünwald Giemsa staining. Scale bar, 10 µm. (B) The purity of the sort was determined at >94% by the staining on the cytospins of sorted cells. More than two hundred cells were counted per cytospin (N=4). (C) Representative immunofluorescence (IF) images of the purified neutrophils from WT or A9$^{-/-}$ animals six hours after *i.p.* injection with BCG are shown. In IF experiments, cells were stained with fluorescence-labeled anti-Ly6G (green) and anti-A9 Abs (purple). Nuclei were counterstained with DAPI (blue). Scale bar, 10 µm.

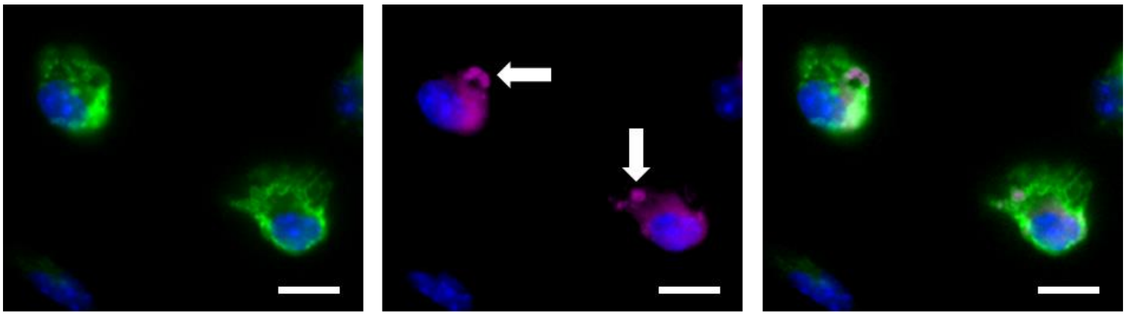

Green: CD68; Purple: CD206; Blue: DAPI

Figure S2. Immunofluorescence of the mouse CD206^{+} M2 macrophages co-cultured with neutrophils, related to Fig. 2. A representative image of the BMMs in the presence of BCG-elicited peritoneal neutrophils is shown. Forty-eight hours after co-culture between BMMs and the neutrophils, the BMMs was stained for CD68 (green), CD206 (purple), and DAPI (Blue). CD68/DAPI (left), CD206/DAPI (middle) and CD68/CD206/DAPI image (right) are shown. CD206 was detected in the cytoplasm to form granular structures (arrows). Bars, 10 µm.

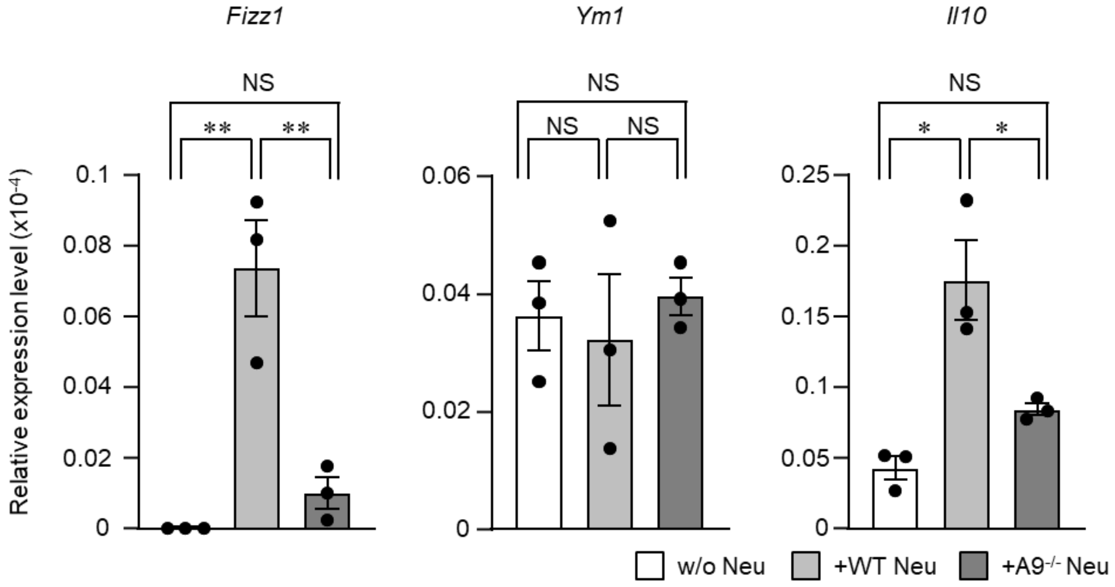

Figure S3. M2 marker levels reduced in the macrophages co-cultured with A9$^{-/-}$ neutrophils compared with those in the macrophages co-cultured with WT neutrophils, related to Fig. 2. BCG-elicited mouse peritoneal neutrophils (WT or A9$^{-/-}$) were co-cultured with BMMs for 48 h in DMEM/10% FCS, collected RNA samples, and analyzed using real-time qRT-PCR. The relative expression levels of Fizz1, Ym1, and IL-10 mRNA were determined using the delta Ct method and normalized with β2m expression levels. All graphs display mean ± SEM. The bar graphs represent one experiment data; each experiment with three mice per group. $^{**}p < 0.005$, $^{*}p < 0.05$, NS, not significant; ANOVA and post hoc Tukey-Kramer test.

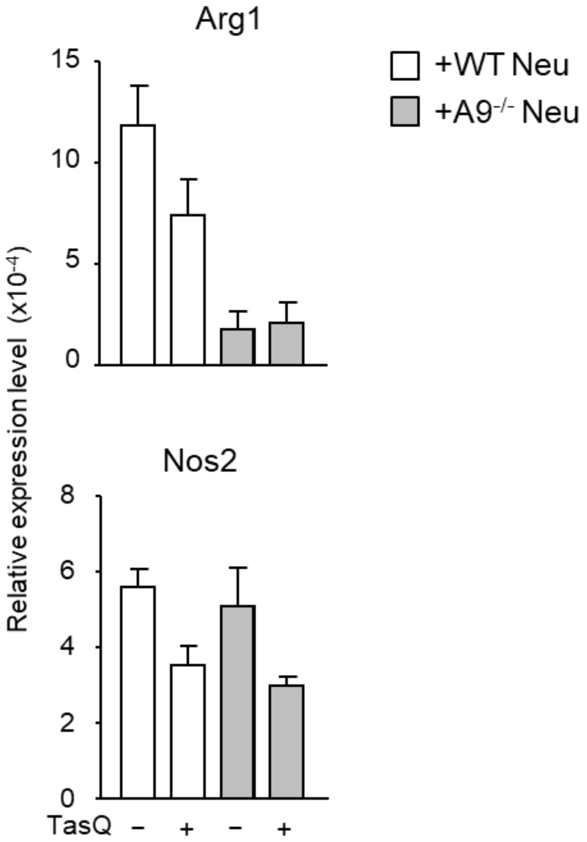

Figure S4. M1 and M2 gene expressions analysis in the BMM-neutrophils co-culture system with or without tasquinimod, related to Fig. 2. The co-culture experiments with WT BMMs and WT or A9$^{-/-}$ Neu were treated with or without tasquinimod (TasQ). The expression of Arg1 and Nos2 mRNAs in the BMMs co-cultured for 48 h. Quantitative RT-PCR values were normalized to the expression levels of the β2-microglobulin gene. The results are from one representative experiment from at least two independent experiments, with four to five mice in each experimental group. Results are shown as the mean ± SEM. n=6 *$p < 0.05$, ANOVA, and post hoc Tukey-Kramer test.

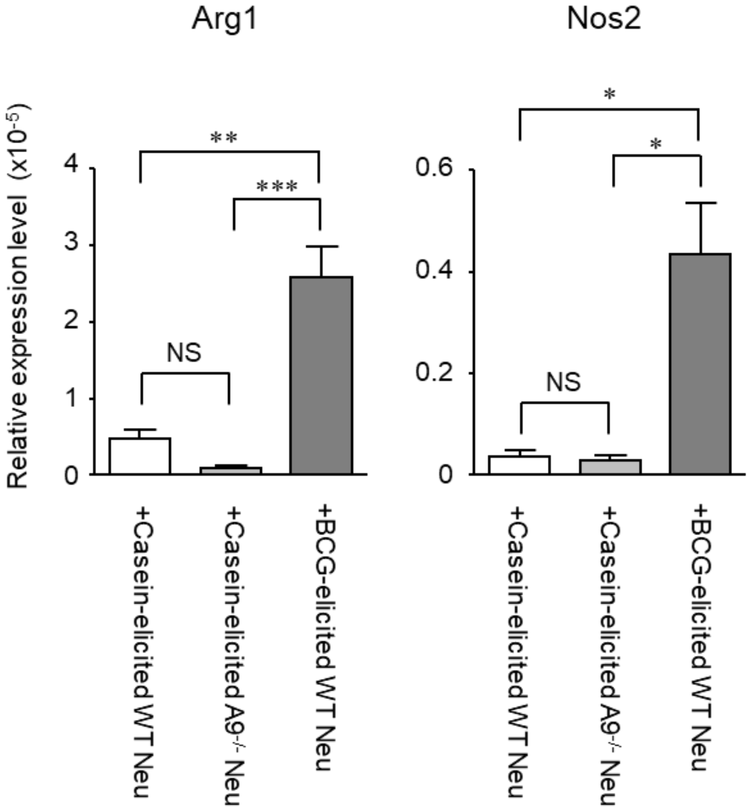

Figure S5. Casein-induced neutrophils could not induce M2 macrophages in the co-culture system, related to Fig. 2. BCG or casein-elicited mouse peritoneal neutrophils (Neu) were co-cultured with BMMs for 48 h in DMEM/10% FCS, collected RNA samples, and analyzed by real-time qRT-PCR. The relative expression level of Arg1 and Nos2 mRNA were determined by the delta Ct method, normalized with β2m expression levels. Results are shown as the mean ± SEM. n=4-8 *$p < 0.05$, ANOVA, and post hoc Tukey-Kramer test.

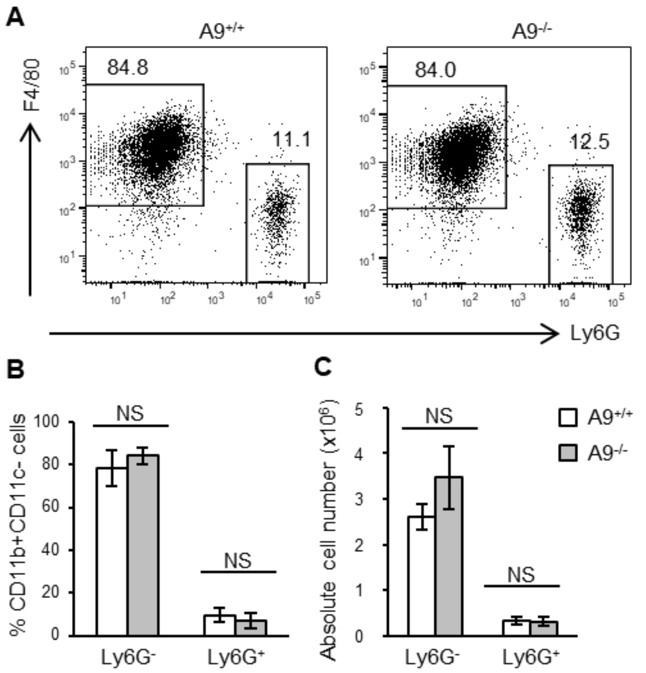

Figure S6. A9$^{-/-}$ Macrophages and neutrophils typically accumulate in the peritoneal cavity after BCG inoculation, related to Fig. 3. (A) Identification of F4/80^{+}Ly6G^{-} macrophages and F4/80^{-}Ly6G^{+} neutrophils in the peritoneal cavity of WT and A9$^{-/-}$ mice injected intraperitoneally with BCG for seven days. Representative FACS plots of F4/80 versus Ly6G on gated CD11b^{+} CD11c^{-} cells are shown in A (the upper panel). (B and C) Frequencies and absolute numbers of these cell populations from WT and A9$^{-/-}$ mice are shown in mean percentages among PI^{-}/singlet/CD11b^{+}/CD11c^{-} populations (B) and mean numbers (C). All graphs display means ± SEM. The bar graphs represent one experiment data from 2 independent experiments, each assessing 3 to 4 mice per group. Differences between the means of the two groups (WT and A9$^{-/-}$) were analyzed by the two-tailed Welch's *t*-test. NS, not a significant difference.

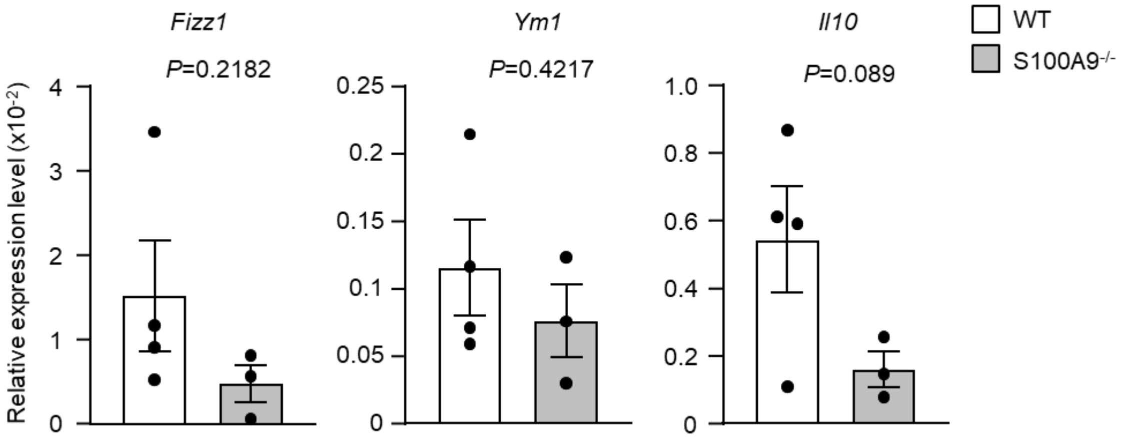

Figure S7. Decreased expression of M2 genes in BCG-injected A9$^{-/-}$ compared with that in WT mice, related to Fig. 3. Five days after BCG inoculation, RNA from the peritoneal macrophages of WT (white) and S100A9$^{-/-}$ (gray) mice was collected and analyzed using qRT-PCR. All graphs display mean ± SEM. The bar graphs represent one experiment data; each experiment with 3–4 mice per group. Differences in the means between the groups (WT and S100A9$^{-/-}$) were analyzed using the two-tailed Welch's *t*-test.

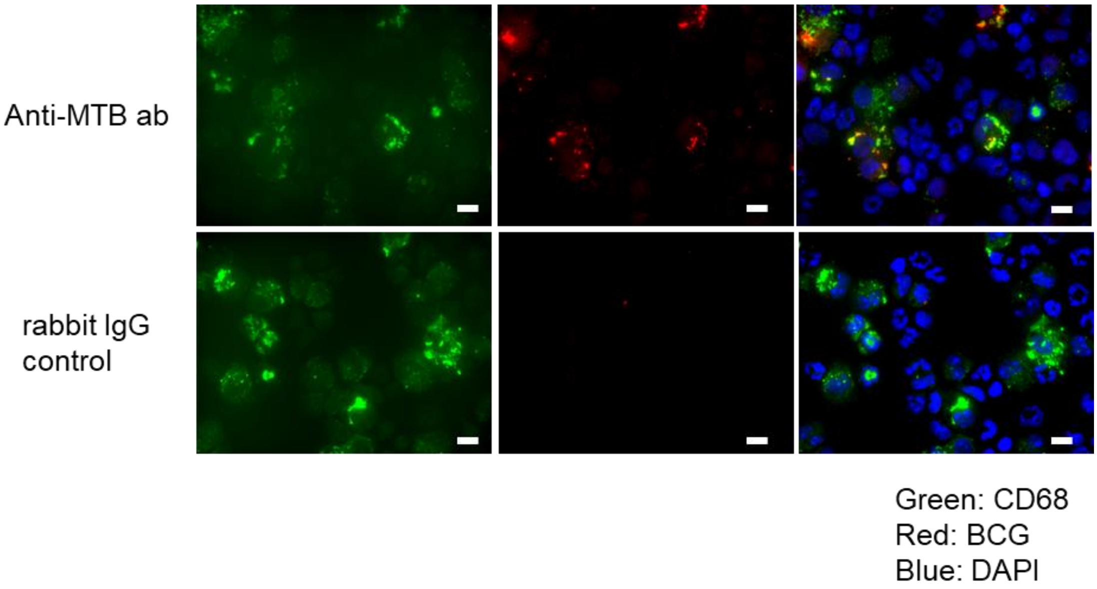

Figure S8. Intracellular BCG in the CD68$^+$ macrophages, related to Fig. 3. (A) Immunofluorescence of peritoneal exudate cells after BCG injection. At one-week post-BCG injection, mouse peritoneal exudate cells were fixed, permeabilized, and stained with anti-CD68 (green), anti-Mycobacterium tuberculosis (MTB, red) (Abcam, #ab905), or rabbit IgG control antibodies. BCG was detected in the cytoplasmic region of CD68$^+$ macrophages. Nuclei stained with DAPI (blue).

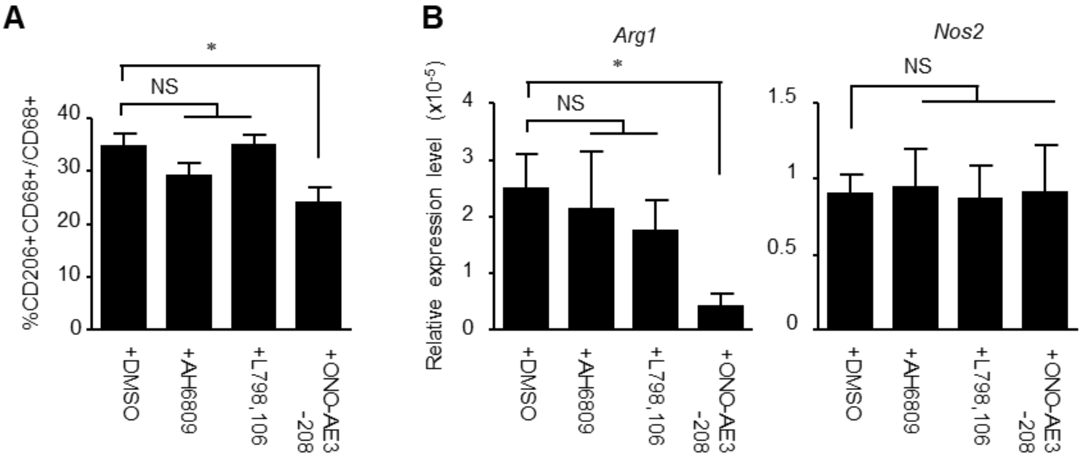

Figure S9. An EP4 antagonist, ONO-AE3-208, specifically inhibited the macrophage M2 polarization controlled by the BCG-elicited neutrophils, related to Fig. 4. (A) BMMs were pre-treated with or without EPs antagonists (AH 6809, L798,106, or ONO-AE3-208), and co-cultured with BCG-elicited Neu for 48 hours in DMEM/10% FCS. Post incubation, cells were fixed and analyzed by immunofluorescence. The percentage of $CD206^{+}$ cells among $CD68^{+}$ total macrophages from WT BMMs treated with or without EPs antagonists is shown. (B) *Arg1* and *Nos2* mRNA induction in BMMs, as indicated in (A), was analyzed via quantitative real-time PCR. Typical data are shown from one experiment out of 3 biological replicates. All graphs display means ± SEM; * $p < 0.05$, one-way ANOVA and post hoc Dunnett's test.

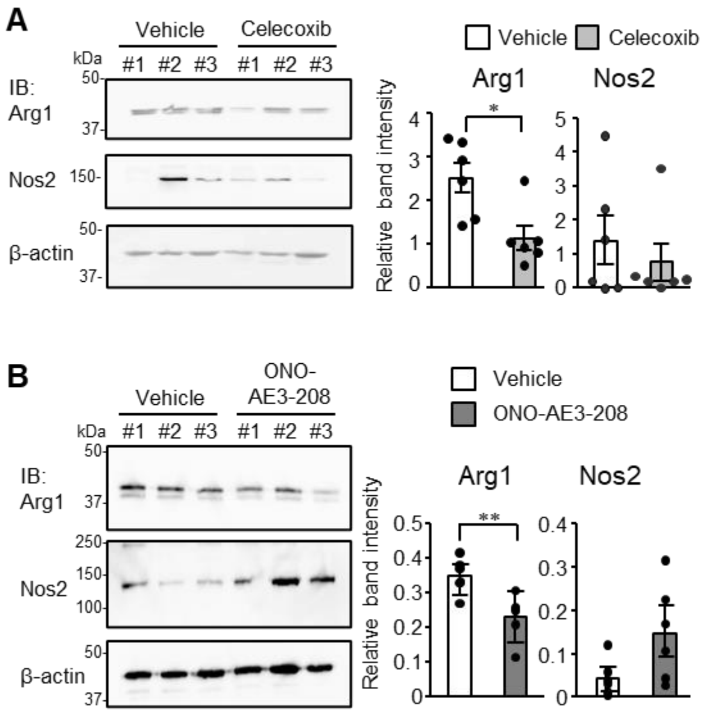

Figure S10. The effect of celecoxib and ONO-AE3-208 in the macrophage polarization *in vivo*, related to Fig. 4. Mice intraperitoneally injected with BCG treated with celecoxib and Vehicle (A), or ONO-AE3-208 and Vehicle (B) for one week. Total cell lysates from these peritoneal macrophages were resolved on SDS-PAGE, and Western blotting was conducted with Abs against Arg1, Nos2, and β-actin. Quantitative data on band intensities were shown in the right panel: Celecoxib (light gray), ONO-AE3-208 (dark gray), and Vehicle (open bars). Immunoblot data were from one representative experiment from at least two independent experiments, with six mice in each experimental group. Data of relative band intensities were pooled from two independent experiments. All results are shown as the mean ± SEM; $^{**}p < 0.005$, $^{*}p < 0.05$, Welch's *t*-test.

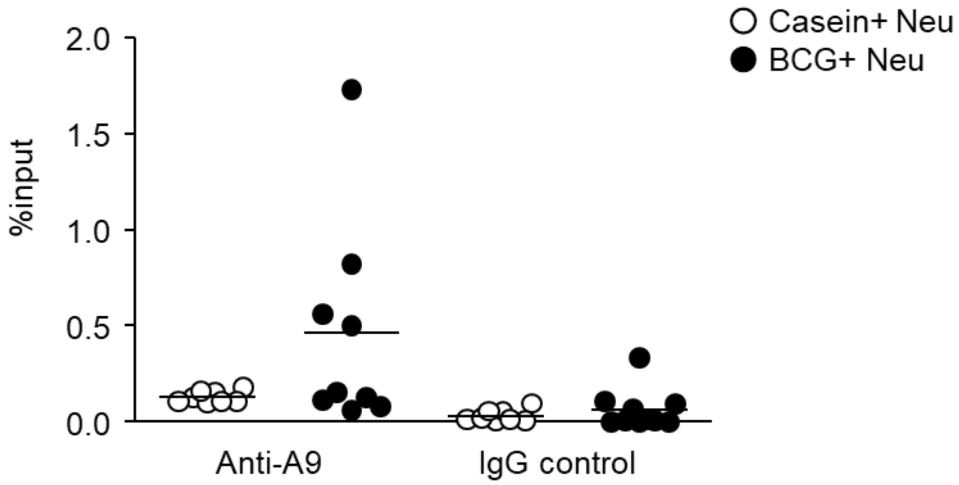

Figure S11. Lacked enrichment on Cox2 promoter in the casein-elicited Neu, related to Fig. 5. ChIP-qPCR assay of the Cox2 gene promoter (region#1, -257 to -121) described in Figure 5D in casein- (○) or BCG-elicited WT Neu (●). A9 was not enriched on promoter region #1 in casein-elicited Neu but the BCG-elicited WT Neu. Data are pooled from two independent experiments.

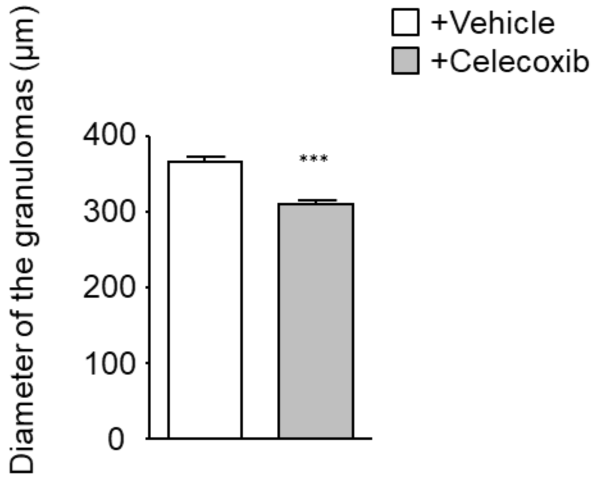

Figure S12. The size of granulomas from celecoxib- and vehicle-treated guinea pigs, related to Fig. 6. The effect of celecoxib on granuloma sizes was evaluated using the guinea pig granuloma model. Lung sections derived from celecoxib-treated (gray) and vehicle-treated (open) guinea pigs were stained with hematoxylin and eosin and measured in diameter using a BZ-X analyzer (Keyence, Osaka, Japan). A bar graph shows the diameters of granulomas (mean ± SEM). ***p < 0.0005, Welch's t-test.

Table S3. Primer sequences used in real-time quantitative RT-PCR, cloning PCR and ChIP qPCR experiments, related to Figs. 2, 4 and 5.

Target	Sequence
Arg1	Forward: TGGCTTGCGAGACGTAGAC
	Reverse: GCTCAGGTGAATCGGCCTTTT
Nos2	Forward: GTTCTCAGCCCAACAATACAAGA
	Reverse: GTGGACGGGTCGATGTCAC
Ptgs2	Forward:TTCAACACACTCTATCACTGGC
	Reverse: AGAAGCGTTTGCGGTACTCAT
Ptges2	Forward: CCTCGACTTCCACTCCCTG
	Reverse: TGAGGGCACTAATGATGACAGAG
Alox15	Forward: GGCTCCAACAACGAGGTCTAC
	Reverse: AGGTATTCTGACACATCCACCTT
Alox5	Forward: TTGCTCTCACAGTATGACTGGT
	Reverse: AGTATCCACGATCTGCTCGAT
Fizz1	Forward: TCCCAGTGAATACTGATGAGA
	Reverse: CCACTCTGGATCTCCCAAGA
Ym1	Forward: CAGGTCTGGCAATTCTTCTGAA
	Reverse: GTCTTGCTCATGTGTGTAAGTGA
Il10	Forward: GCTCTTACTGACTGGCATGAG
	Reverse: CGCAGCTCTAGGAGCATGTG
β2-microglobulin	Forward: TTCTGGTGCTTGTCTCACTGA
	Reverse: CAGTATGTTCGGCTTCCCATTC
Cox2 promoter #1	Forward: GGTTCTTGCGCAACTCACTG
	Reverse: ACTGGCTGCTAATGGGGAGA
Cox2 promoter #2	Forward: TAGCTGTGTGCGTGCTCTGA
	Reverse: AGGGAATCCCCTCACCTCTC
Mouse A9 cloning (XhoI-NotI)	Forward: ATCCTCGAGATGGCCAACAAAGCACCTTCTCA
	Reverse: CATGCGGCCGCTTACTTCCCACAGCCTTTGC
Mouse C/EBPβ cloning	Forward: ATGCCTCGAGATGCACCGCCTGCTGGCCTGGGACGC
	Reverse: CATGAATTCGCAGTGGCCCGCCGAGGCCAGCAGCGGC
